# Distinct Gut–Brain Axis Dysregulation in Episodic Versus Chronic Migraine: Insights from NTG-Induced Mouse Models

**DOI:** 10.3390/ijms262110493

**Published:** 2025-10-29

**Authors:** Dae-Chul Shin, Harry Jung, Songyi Park, Dan-Gyeong Song, Sang-Hwa Lee, Jong-Hee Sohn

**Affiliations:** 1 Department of Biomedical Science, Hallym University, Chuncheon 24252, Republic of Korea; bill916@naver.com (D.-C.S.); songdg18@gmail.com (D.-G.S.); 2 Institute of New Frontier Research Team, College of Medicine, Hallym University, Chuncheon 24252, Republic of Korea; harry_88@naver.com (H.J.); songyip3697@gmail.com (S.P.); bleulsh@naver.com (S.-H.L.); 3 Department of Neurology, Chuncheon Sacred Heart Hospital, Hallym University College of Medicine, Chuncheon 24252, Republic of Korea

**Keywords:** nitroglycerin, episodic migraine, chronic migraine, calcitonin gene-related peptide, gastrointestinal tract

## Abstract

The gut–brain axis regulates brain functions and maintains central nervous system homeostasis and intestinal balance. Migraine patients often present with gastrointestinal (GI) comorbidities, with stronger associations observed in chronic migraine (CM) than in episodic migraine (EM). To investigate migraine-related GI alterations, nitroglycerin (NTG)-induced mouse models of EM (N = 15) and CM (N = 15) were established using single or repeated NTG injections (10 mg/kg). EM mice were euthanized 4 h after a single injection, whereas CM mice received NTG every other day for 9 days and were euthanized after the fifth injection. On the day of sacrifice, GI tissues were analyzed for morphological changes, cytokine expression, calcitonin gene-related peptide (CGRP) levels, and immune cell profiles. NTG-treated groups exhibited significant reductions in both food intake and body weight compared with controls. In addition, colon length was markedly shortened in the chronic migraine (CM) model (*p* < 0.01). Molecular analyses revealed distinct cytokine expression profiles between models: the episodic migraine (EM) model showed increased levels of proinflammatory cytokines (IL-1β, IL-6, and IL-8; *p* < 0.01), whereas the CM model displayed elevated anti-inflammatory cytokines (IL-4, IL-10, and TGF-β; *p* < 0.01), particularly in the colon. CGRP expression was also markedly upregulated throughout the gastrointestinal tract, with the highest expression observed in the colon of CM mice (*p* < 0.01). Flow cytometric immune profiling further demonstrated divergent immune cell patterns, with increased Th17 (*p* = 0.0085) and B cell (*p* = 0.0199) populations in EM, while CM was characterized by enrichment of T cells (*p* = 0.0221), regulatory T cells (Tregs) (*p* = 0.0114), and macrophages (*p* = 0.0062), indicating more pronounced immune alterations in the distal colon. These findings indicate that CM involves more severe gut–brain axis dysregulation than EM, supporting the potential of gut-targeted therapies as adjunct strategies in chronic migraine.

## 1. Introduction

Migraine is a prevalent neurological disorder that causes substantial episodic disability and reduced productivity [1,2,3,4]. It is marked by recurrent episodes of moderate to severe headaches, frequently accompanied by classical symptoms including nausea and vomiting. It is also often complicated by gastrointestinal (GI) conditions [5,6,7]. GI symptoms have been included in the migraine diagnostic criteria based on the International Classification Headache Disorders, and the American Headache Society recommends non-oral therapies for patients with GI symptoms [8]. Previous studies showed that migraine is associated with GI disorders as comorbidities, as well as overlapping symptomatology, including *Helicobacter pylori* infection, gastroesophageal reflux disorder, irritable bowel syndrome, gastroparesis, hepatobiliary disorders, and celiac disease [9,10]. Although the pathophysiology of migraine is not fully understood, this association suggests a relation with the GI tract, suggesting a pathophysiology involving the gut–brain axis. While rates of migraine headache comorbidities generally increase with increases in migraine headache frequency, the rate of stomach/intestinal ulcers is approximately double in cases of chronic migraine (CM) compared to episodic migraine (EM) [11,12].

Among the various migraine experimental models available, the nitric oxide (NO)-induced migraine model, particularly nitroglycerin (NTG)-induced migraine, has been widely utilized to mimic the clinical features of both EM and CM [13,14]. NTG, an NO donor, induces migraines by triggering vasodilation and activating inflammatory pathways, leading to prolonged sensitization of pain-processing structures [15,16].

Clinical and experimental data suggest that migraines are frequently associated with GI disturbances, including altered gut motility, inflammation, and changes in the composition of the gut microbiota [17]. However, the underlying mechanisms linking migraines to GI dysfunction or disorders, particularly the differential pathways distinguishing EM and CM, remain unclear [18,19]. While previous studies have indicated acute inflammatory gut alterations following NTG administration, a comprehensive and integrated analysis of the full spectrum of morphological, neuropeptidergic, and cellular immunological changes in both EM and CM models is lacking [20,21]. Given that inflammation plays a crucial role in the pathophysiology of migraine, investigating the stage-dependent inflammatory and cellular responses in the GI tract may provide new insights into migraine-associated systemic effects [22,23].

In this study, NTG-induced EM and CM mouse models were established to explore the impact of migraine on the gastrointestinal tract through an integrated, multi-parametric analysis of cytokine expression, calcitonin gene-related peptide (CGRP) levels, and immune cell redistribution, thereby defining the distinct gut–brain axis signatures associated with EM and CM. By identifying molecular and cellular changes in the GI tract under migraine conditions, this study enhanced our understanding of the role of the gut–brain connection in the pathophysiology of migraine.

## 2. Results

### 2.1. Changes in the GI Tract in NTG-Induced EM and CM Models

**Figure 1:**
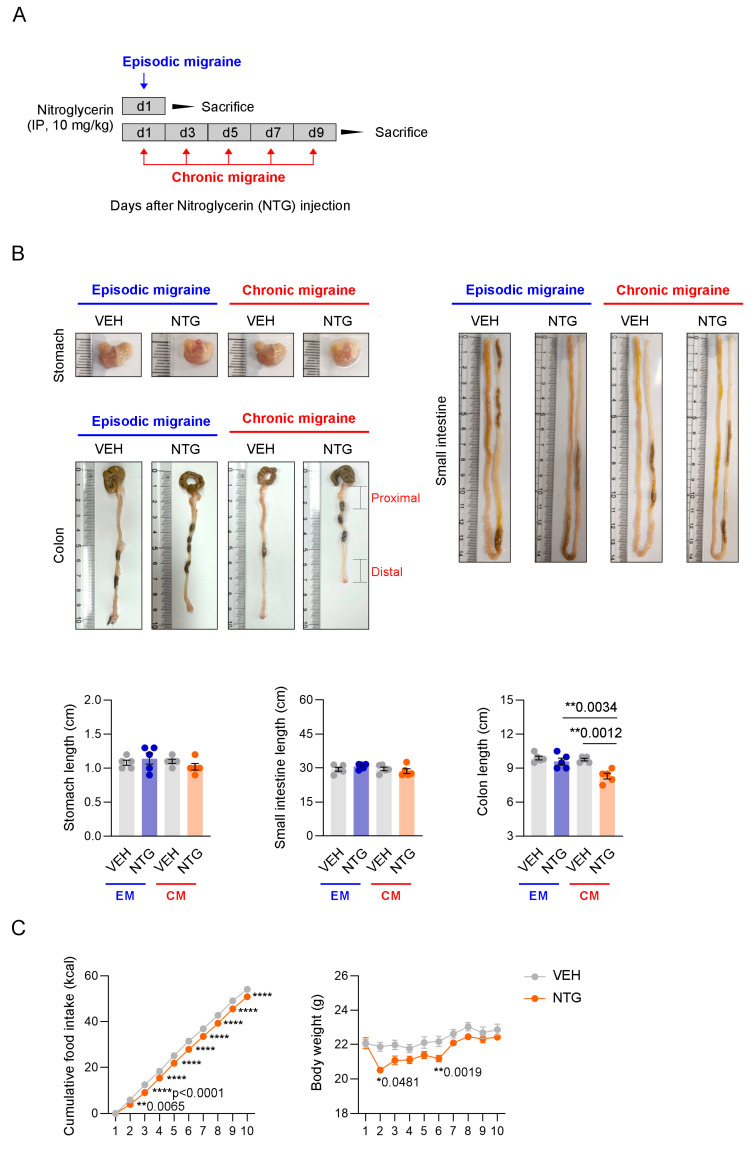
Functional and morphological changes in the GI tract in NTG-induced migraine models. (**A**) Experimental protocol for analysis of the gastrointestinal (GI) tract in NTG-induced episodic migraine (EM) and chronic migraine (CM) models. (**B**) Macroscopic comparison of the stomach, small intestine, and colon in NTG-induced migraine models. Quantification of stomach (N = 5), small intestine (N = 5), and colon (N = 5) lengths in NTG-induced migraine models. Results are presented as mean ± SEM. One-way ANOVA with Tukey’s multiple comparisons test: ** *p* < 0.01. (**C**) Time course measurement of cumulative food intake (N = 5) and body weight (N = 5) over 10 days in NTG-induced migraine models. Results are presented as mean ± SEM. Two-way ANOVA with Sidak’s multiple comparisons test: * *p* < 0.05; ** *p* < 0.01; **** *p* < 0.0001. IP, intraperitoneal; NTG, nitroglycerin; VEH, vehicle control.

To analyze the GI tract in established EM and CM models associated with NTG administration, the lengths of the stomach, small intestine, and colon were examined. Comparison of GI tract length between the EM and CM models revealed a difference in colon length, but not in the stomach or small intestine length. Quantitative analysis of the length of each part of the GI tract revealed significant shortening of the colon in the NTG-induced CM model compared to the EM model and controls (Figure 1B; Supplementary Figure S1). To determine whether NTG-induced migraine affects GI motility, we indirectly analyzed changes in food intake and body weight in mice. Interestingly, both the food intake and body weight of mice were significantly reduced in the NTG-induced migraine groups compared to the vehicle controls (Figure 1C; Supplementary Figure S1). These results suggest that NTG-induced migraine induced morphological and functional changes in the overall GI tract, with significant changes in colon length in the CM model.

### 2.2. Differences in Inflammatory Cytokines in the GI Tract Between NTG-Induced EM and CM Models

**Figure 2:**
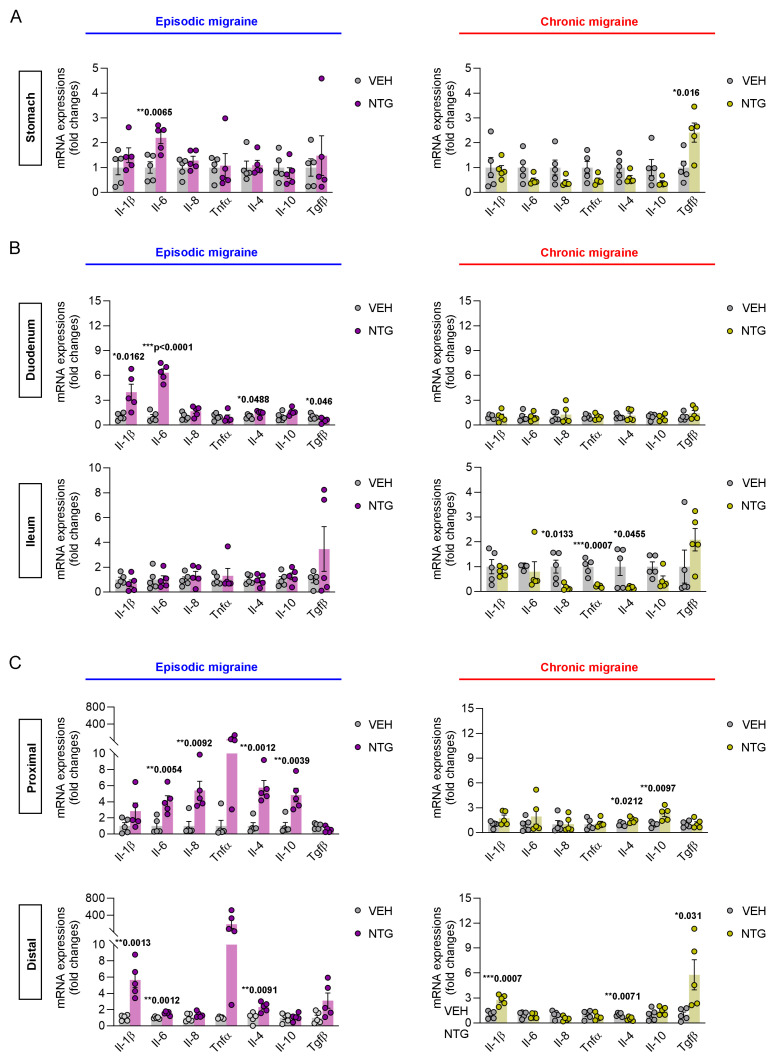
Differential inflammatory cytokine expression in the stomach, small intestine, and colon of NTG-induced EM and CM models. (**A**) qRT-PCR analysis of cytokine expression (IL-1β, IL-6, IL-8, TNF-α, IL-4, IL-10, and TGF-β) in the stomach of EM (N = 5) and CM (N = 5) mouse models. (**B**) qRT-PCR analysis of inflammatory cytokines in the duodenum and ileum of NTG-induced EM (N = 5) and CM (N = 5) models. (**C**) qRT-PCR analysis of inflammatory cytokines in the proximal and distal colon of NTG-induced EM (N = 5) and CM (N = 5) models. (**A**–**C**) Results are presented as mean ± SEM. Statistics were calculated using unpaired Student’s *t*-test: * *p* < 0.05; ** *p* < 0.01; *** *p* < 0.001. NTG, nitroglycerin; VEH, vehicle control.

To analyze the molecular biological differences in the GI tract between the NTG-induced EM and CM models, qRT-PCR was performed to examine the expression levels of inflammatory cytokines in the stomach, small intestine, and colon. Interestingly, qRT-PCR analysis of GI tissues showed contrasting results between the NTG-induced EM and CM mouse models. In the stomach, the level of interleukin (IL)-6 expression was significantly increased in the EM model, whereas the level of transforming growth factor (TGF)-β expression was significantly elevated in the CM model (Figure 2A; Supplementary Figure S2). In the duodenum, the expression levels of cytokines such as IL-1β, IL-6, IL-4, and TGF-β were significantly increased in the EM model, while no significant differences were observed in the CM model compared to the vehicle control group. In contrast, in the ileum, no significance differences in cytokine levels were observed in the EM model, while the expression levels of cytokines such as IL-8, tumor necrosis factor [TNF]-α, and IL-4 were significantly higher in the CM model compared to the controls (Figure 2B; Supplementary Figure S3). In addition, the colon was divided into proximal and distal regions for inflammatory cytokine expression analysis. In the proximal colon, the expression levels of pro- and anti-inflammatory cytokines such as IL-6, IL-8, IL-4, and IL-10 were significantly increased in the EM model, while the CM model showed increased expression of IL-4 and IL-10. Furthermore, in the distal colon, IL-1β, IL-6, and IL-4 were highly expressed in the EM model, while IL-1β, IL-4, and TGF-β were highly expressed in the CM model (Figure 2C; Supplementary Figure S4). Taken together, these results show that different types of inflammatory cytokines were expressed in the GI tracts of NTG-induced EM and CM models.

### 2.3. Differences in CGRP Expression in the GI Tract Between NTG-Induced EM and CM Models

**Figure 3:**
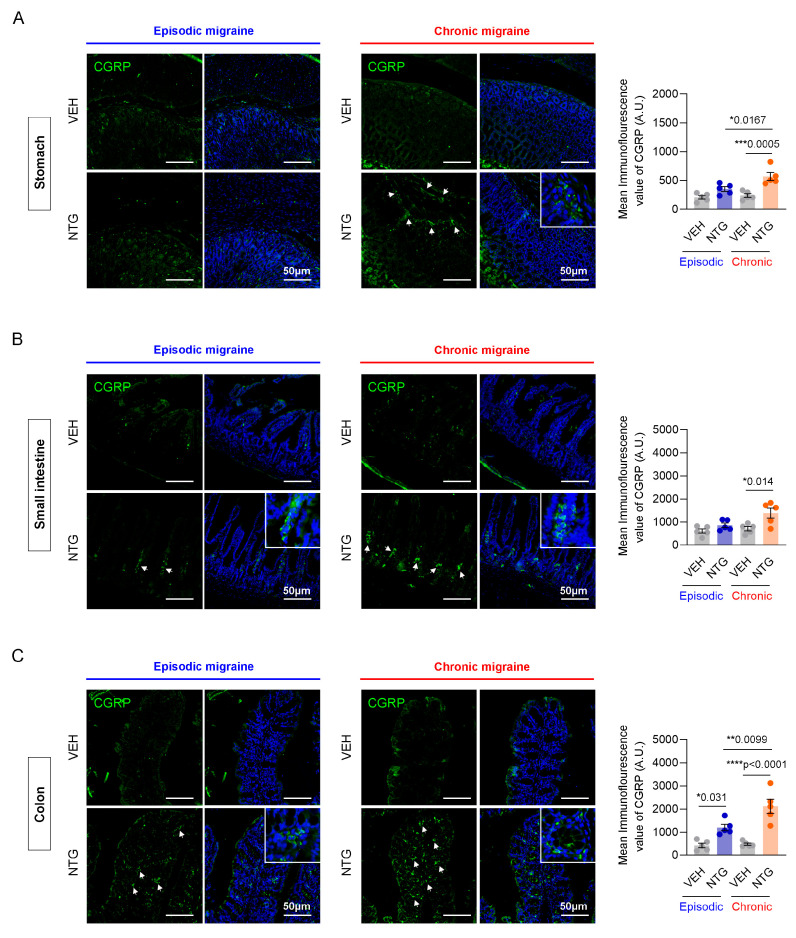
Immunofluorescence analysis of CGRP levels in the stomach, small intestine, and colon of NTG-induced EM and CM models. (**A**) Representative images of CGRP immunofluorescence staining in the stomach of EM (N = 5) and CM (N = 5) models. (**B**) Representative images of CGRP immunofluorescence staining in the small intestine of EM (N = 5) and CM (N = 5) models. (**C**) Representative images of CGRP immunofluorescence staining in the colon of EM (N = 5) and CM (N = 5) models. (**A**–**C**) Green, CGRP staining; blue, DAPI staining, Scale bars = 50 μm. Results are presented as mean ± SEM. Statistical analysis via one-way ANOVA with Tukey’s multiple comparisons test: * *p* < 0.05; ** *p* < 0.01; *** *p* < 0.001; **** *p* < 0.0001. NTG, nitroglycerin; VEH, vehicle control.

Immunofluorescence staining was performed to evaluate the differential expression patterns of CGRP in the gastrointestinal tract of NTG-induced episodic and chronic migraine mouse models, specifically within the stomach, small intestine, and colon. In the stomach, CGRP levels showed a slight increase in the EM model compared to controls, but the difference did not reach statistical significance. However, in the CM model, CGRP expression was significantly increased compared with the EM model and the control group (Figure 3A; Supplementary Figure S5). In the small intestine, there was no significant difference between the EM and control group. In contrast, CGRP expression in the CM model was significantly increased compared with controls (Figure 3B; Supplementary Figure S5). In the colon, CGRP expression was significantly increased in both NTG-induced EM and CM models, with a significant increase seen in the CM group compared to the EM group (Figure 3C; Supplementary Figure S5). Our results show that CGRP expression in the GI tract was increased in NTG-induced migraine models, and the effect was more pronounced in the CM model, particularly in the colon.

### 2.4. Differences in Immune Cell Responses in the Colon Between NTG-Induced EM and CM Models

**Figure 4:**
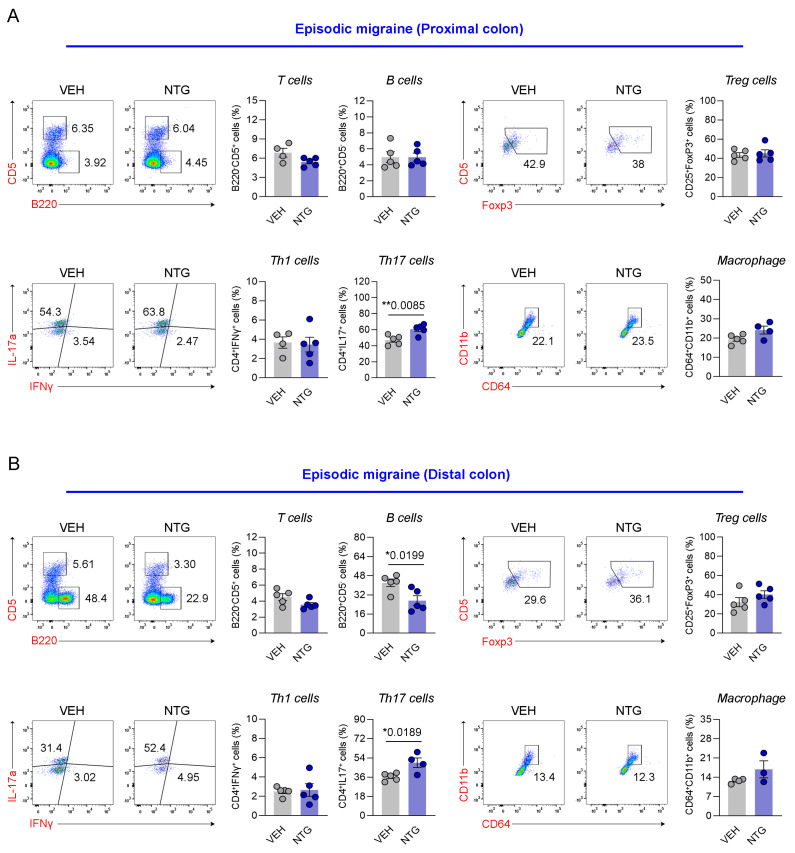
Flow cytometric analysis of colonic immune cell subsets in the NTG-induced EM model mice. (**A**) Flow cytometric characterization of T cells, B cells, Tregs, Th subsets, and macrophages in the proximal colon of the EM (N = 4–5) model. (**B**) Flow cytometric characterization of T cells, B cells, Tregs, Th subsets, and macrophages in the distal colon of the EM (N = 3–5) model. Results are presented as mean ± SEM. Statistics were calculated using unpaired Student’s *t*-test: * *p* < 0.05; ** *p* < 0.01. NTG, nitroglycerin; VEH, vehicle control.

**Figure 5:**
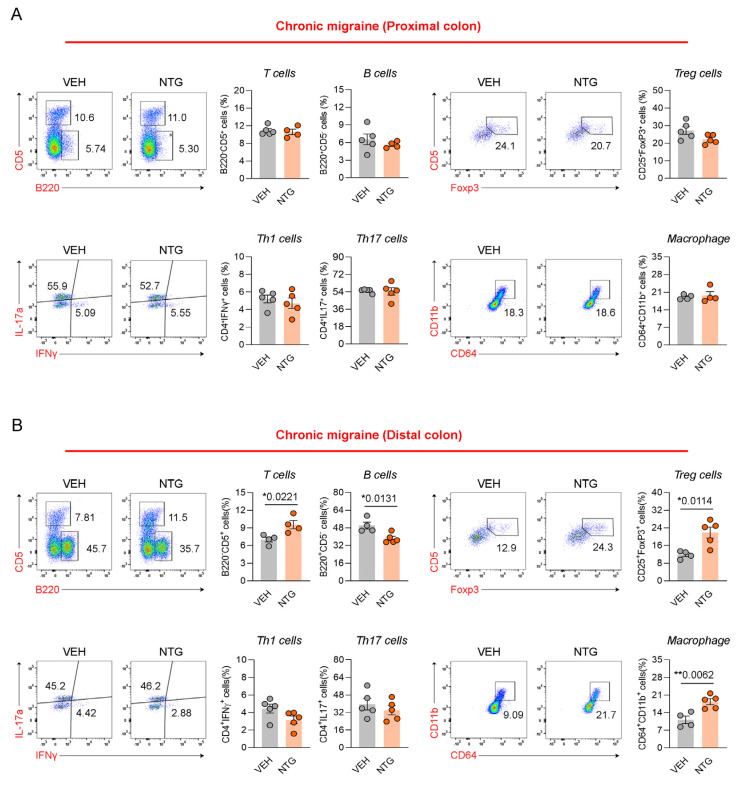
Flow cytometric analysis of colonic immune cell subsets in NTG-induced CM model mice. (**A**) Flow cytometric characterization of T cells, B cells, Tregs, Th subsets, and macrophages in the proximal colon of CM (N = 4–5) model. (**B**) Flow cytometric characterization of T cells, B cells, Tregs, Th subsets, and macrophages in the distal colon of the CM (N = 4–5) model. Results are presented as mean ± SEM. Statistics were calculated using unpaired Student’s *t*-test: * *p* < 0.05; ** *p* < 0.01. NTG, nitroglycerin; VEH, vehicle control.

Our results show that the NTG-induced migraine model is associated with morphological and functional changes in the GI tract, with particularly high levels of inflammatory cytokines and CGRP expression observed in the colon. Therefore, we analyzed the distribution of immune cells in the colon tissue via FACS analysis. The distribution of immune cells was determined in the proximal and distal colon of mice through the analysis of T cells, B cells, Th1 cells, Th17 cells, regulatory T (Treg) cells, and macrophages. In the EM model, Th17 cells were significantly increased in the proximal colon, while there were no significant differences in T cells, B cells, Th1 cells, Treg cells, or macrophages (Figure 4A; Supplementary Figure S6). In addition, B cells and Th17 cells were significantly increased in the distal colon of the EM model, while T cells, Th1 cells, Treg cells, and macrophages showed no significant changes (Figure 4B; Supplementary Figure S6). In the proximal colon of the CM model, no significant differences were observed in any immune cell population, including T cells, B cells, Th1 cells, Th17 cells, Treg cells, and macrophages (Figure 5A; Supplementary Figure S6). In the distal colon of the CM model, various immune cells, including T cells (excluding Th1 and Th17 cells), Treg cells, and macrophages, were significantly increased, whereas B cells were markedly decreased (Figure 5B; Supplementary Figure S6). Our findings show that the immune cell distribution in the CM model was different from the EM model, with elevated numbers of Th17 and B cells in the EM model, and of T cells, Treg cells, and macrophages in the CM model.

## 3. Discussion

**Figure 6:**
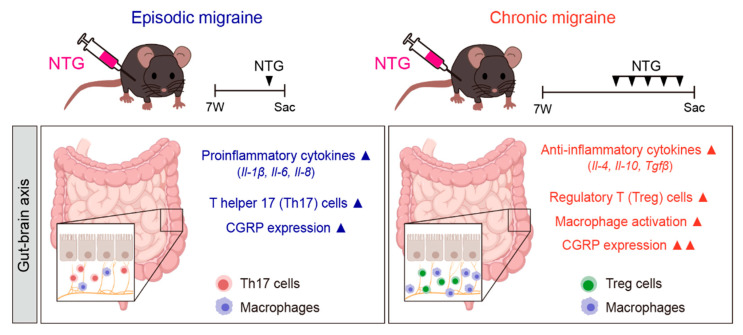
Summary of the NTG-induced migraine model and associated gastrointestinal changes. EM was induced through a single intraperitoneal injection of NTG (10 mg/kg), while CM was induced via repeated NTG administration on days 1, 3, 5, 7, and 9. The diagram illustrates how migraine alters the GI tract through the gut–brain axis. EM was associated with elevated proinflammatory cytokines (IL-1β, IL-6, and IL-8), Th17 cell activation, and mild CGRP expression. In contrast, CM was characterized by increased anti-inflammatory cytokines (IL-4, IL-10, and TGF-β), enhanced regulatory T cell and macrophage activity, and robust CGRP expression in the GI tract. ▲ indicates upregulation (increase). Images were created using BioRender.com.

In this study, we investigated gut-related morphological, molecular, and immunological alterations in NTG-induced EM and CM mouse models, with a particular focus on the colon as a representative site of gut–brain axis involvement. CM mice showed greater GI alterations than EM mice, with the most prominent changes being observed in the colon of the CM model, including morphological shortening, altered cytokine profiles, increased CGRP expression, and immune cell redistribution. These findings support the suggestion that CM is associated with a profound disruption of gut–brain axis homeostasis (Figure 6).

Previous reports suggest a close association between migraine and GI disorders, with individuals with migraine showing a higher likelihood of developing conditions such as constipation, diarrhea, dyspepsia, irritable bowel syndrome, and gastroesophageal reflux disease [24,25,26]. Therefore, it has been postulated that migraine may affect the gut–brain axis, but the precise mechanism underlying this relation remains unclear. A variety of factors have been suggested to influence the gut–brain interaction, including inflammatory mediators (IL-1β, IL-6, IL-8, TNF-α), the gut microbiota profile, neuropeptide and serotonin pathways, stress hormones, and dietary components [27]. Previous studies showed increased immune cell infiltration and inflammation in the colonic tissue 4 h after a single injection of NTG [20,21]. NTG-induced migraine models are widely used to study both EM and CM due to their ability to replicate clinical features [28,29,30]. In this study, to examine changes in the GI tract induced by migraine, we established EM and CM mouse models by administering NTG according to different schedules. Phenotypic differences in the GI tract that were dependent on the duration of migraine induction by NTG were detected. A morphological comparison of the stomach, small intestine, and colon tissues revealed specific shortening of the colon in the CM model. In addition, indirect analysis to assess the impact of the migraine model on GI motility revealed significant decreases in food intake and body weight in the NTG-induced model compared to controls [31,32,33,34]. The phenotypic changes observed in our study, such as the shortening of the intestinal length and decreased GI motility, were consistent with clinical observations. Mechanistically, the NO donor NTG initiates central sensitization and inflammation in the trigeminovascular system, but its peripheral effects on the gut are significant. NO, which is released directly in the brain in response to excitation and activation of trigeminal neurons, can affect the GI tract through an indirect mechanism when it crosses the blood–brain barrier (BBB) and reaches the gut. Furthermore, this central NO signaling triggers the systemic release of inflammatory mediators and neuropeptides, which then modulate the enteric nervous system (ENS) and mucosal immunity, influencing GI hormones, motility, and appetite regulation [35].

Previous reports, demonstrated acute NTG-induced mast cell inflammation and increases in proinflammatory cytokines like TNF-α and IL-1β in the colon, providing critical groundwork for GI involvement in migraine [21,28,36]. Our study significantly advances these findings by providing the first comprehensive comparison of EM and CM gut–brain axis signatures, using a uniquely integrated, multi-parametric approach. To perform a more complete and stage-dependent analysis of GI tissues than previous studies, we examined morphological shortening, CGRP expression, and detailed immune cell subsets via FACS, in addition to region-specific qRT-PCR. From a molecular perspective, differential patterns of inflammatory cytokine expression were observed throughout the GI tract, with the EM model showing predominantly proinflammatory responses (e.g., IL-6, IL-1β, and IL-8), while the CM model was characterized by increased expression of anti-inflammatory mediators (IL-4, IL-10, and TGF-β), particularly in the colon. We acknowledge that classifying IL-10 and TGF-β as purely “anti-inflammatory” may oversimplify their complex, context-dependent roles; TGF-β, for example, is critical for tissue remodeling and can have pro-fibrotic effects, while IL-10’s function varies based on cellular context. However, the increase in these cytokines alongside the elevation of Treg cells and macrophages in the CM distal colon strongly suggests the activation of immune regulatory circuits or an adaptive immunomodulatory response following prolonged or repeated neurogenic inflammation, which is consistent with known mechanisms of tolerance or exhaustion pathways often mediated by IL-10 and TGF-β under chronic stimulation. This CM-specific gut immune remodeling may result from a potential causal pathway: repeated NTG exposure leads to sustained peripheral CGRP release, which, combined with systemic inflammation, continuously challenges the enteric immune system. This chronic challenge then drives the ENS and mucosal immune cells to switch from an acute proinflammatory state to a state of sustained immune tolerance and tissue preservation, characterized by the enrichment of regulatory cells. This finding significantly extends beyond the acute inflammatory changes previously reported [20,21].

CGRP is widely expressed in various organ systems, including the nervous, cardiovascular, digestive, respiratory, genitourinary, and musculoskeletal systems, where it is primarily involved in vasodilation and nociceptive modulation [37,38,39]. In particular, CGRP plays a pivotal role in the nervous, cardiovascular, and digestive systems, and is critically involved in the regulation of inflammatory processes [40,41,42]. Elevated CGRP expression in the GI tract, particularly in the colon of CM mice, further highlights the involvement of this neuropeptide not only in central trigeminovascular pathways but also in the peripheral enteric system. CGRP is known to influence gut motility, mucosal immunity, and vascular tone [43], and its persistent elevation in CM may contribute to altered GI function and immune signaling, thereby reinforcing gut–brain axis dysregulation Crucially, this robust peripheral CGRP signal provides strong experimental support for the observed clinical link between CM severity and GI comorbidities, as clinical evidence indicates that CM patients often report more severe and frequent gastrointestinal symptoms compared to EM patients. Furthermore, human studies have demonstrated associations between altered gut microbiota composition and headache frequency, as well as distinct immune activation patterns in people with migraines, providing clinical context for the immune and neuropeptide shifts observed in our model [8,27].

To validate these findings, the distribution of immune cells was examined via FACS analysis of colonic tissues, which showed significant differences in immune cell responses between the EM and CM models. FACS analysis revealed divergent immune cell distributions in the colon. In the EM model, increased Th17 and B cell populations suggested an active mucosal immune response. In contrast, the CM model showed increased T cells, Treg cells, and macrophages, particularly in the distal colon, an area increasingly recognized as a site of intense neuroimmune interaction. These differences in immune landscape between EM and CM may reflect stage-dependent immunomodulatory responses to repeated NTG stimulation, and may be relevant to the clinical transition from EM to CM [13].

The findings of the present study lend experimental support to this bidirectional model, in which persistent central sensitization and neurogenic inflammation modulate gut immunity and motility, while gut-derived inflammatory or neuropeptidergic signals may, in turn, affect trigeminal system excitability. From the perspective of translational research, these results highlight the potential of targeting gut-derived pathways in the management of migraine. Microbiome-based therapies, dietary modulation, probiotics, and mucosal immunotherapy are emerging areas of interest, particularly given recent evidence linking altered gut microbiota composition to headache frequency and severity [27,44]. The significant peripheral CGRP upregulation in CM strongly supports the efficacy of systemic CGRP-targeting therapeutics (e.g., monoclonal antibodies or gepants) that are known to act on peripheral sites [45]. Furthermore, the CM-specific shift toward an adaptive immune state observed in our model raises an intriguing translational question: Could this unique chronic gut immune environment locally influence the efficacy or pharmacodynamics of systemic CGRP-targeting drugs? The local cytokine milieu might alter CGRP receptor expression or downstream signaling in the enteric nervous system, thereby modulating the therapeutic outcome. Given the importance of the gut–brain connection in migraine pathogenesis, the observed immune cell changes and our findings also advocate for gut-targeted interventions, such as dietary modulation or microbiota-based therapies [8], as crucial adjunct strategies to complement pharmacological treatments.

Nonetheless, this study had several limitations. First, while the NTG model recapitulates certain aspects of migraine pathophysiology, it may not fully represent the heterogeneity of human migraine, particularly with respect to underlying genetic or environmental factors. We also acknowledge that the absence of parallel behavioral or pain endpoints (such as grimace scores or assessments of photophobia/phonophobia) is a limitation. This lack of concurrent data weakens the ability to directly correlate the magnitude of the observed GI morphological, molecular, and immune changes with the severity of the migraine-like phenotype, thus reducing direct translational relevance. Further studies integrating comprehensive GI analysis with detailed pain and behavioral assessments are required to clarify these direct correlations. Second, the observed cytokine and immune cell changes were limited to bulk tissue analysis and flow cytometry. Future studies using single-cell transcriptomics or spatial profiling could offer higher-resolution insights into cell type-specific alterations. Third, gut microbiota composition was not evaluated in this study, despite its known influence on immune tone and neuropeptide expression. We acknowledge that the omission of gut microbiota profiling (16S rRNA or metagenomics) is a major limitation of this study, especially since disruption of the gut–brain axis is a central claim. While we focused on host immunity and neuropeptide signaling, the pronounced alterations we observed—including the differential activation of Th17/B cells in EM versus Treg/macrophages in CM, alongside changes in anti-inflammatory cytokines—strongly suggest an underlying dysbiosis. Given the known link between the microbiome, immune tone, and neuropeptide expression (such as CGRP), integrating microbiome profiling with our host immune and neuronal signaling analyses in future work is essential to fully contextualize these findings and is a crucial next step. Finally, a total of fifteen mice were used per group. However, to accommodate methodological requirements and ensure assay-specific precision, animals were subdivided into subsets of five for qRT-PCR, FACS, and immunofluorescence analyses, respectively. This design enabled the collection of high-quality tissue and cell samples from defined intestinal regions optimized for each technique, thereby minimizing technical variability across assays. Although the effective sample size per assay was limited, preliminary experiments confirmed consistent and reproducible results under standardized conditions. We recognize that the relatively small sample size may reduce statistical power and increase the likelihood of type II errors; therefore, future studies with larger cohorts are planned to validate and extend these findings.

Overall, our findings provide experimental evidence that CM is associated with more extensive gut–brain axis disruption than EM, characterized by GI inflammation, CGRP expression, immune cell alterations, and morphological changes in the colon. These results highlight the gut as a potential therapeutic target in migraine, especially CM, and encourage further translational research to explore microbiota-based therapies, mucosal immune modulators, and peripheral CGRP-targeting interventions as disease-modifying strategies.

## 4. Materials and Methods

### 4.1. Animals

All animal experiments were approved by the Institutional Animal Care and Use Committee (IACUC) of Hallym University (Hallym #2023-81, approved on 19 February 2024, Chuncheon, Republic of Korea). Male C57BL/6J mice (7 weeks old, 21.0 ± 1.0 g) were purchased from DooYeol Biotech Korea under SPF conditions. Mice were housed, grouped, and acclimatized with food and water ad libitum under a 12 h light/dark cycle (8:00 a.m. to 8:00 p.m.) at 23 °C ± 2 °C and 55% ± 10% humidity. Although migraine is more prevalent in females, this study exclusively utilized male mice to eliminate potential variability arising from female hormonal fluctuations [28].

### 4.2. NTG-Induced Migraine Model

NTG was synthesized as described previously [28] and prepared from 200 mg of 0.974% solution in propylene glycol (Cat No. 1466506; USP, Rockville, MD, USA). For all in vivo experiments, NTG was freshly diluted in 100 μL of saline and administered intraperitoneally at a dose of 10 mg/kg. The vehicle control in these experiments consisted of the same amount of propylene glycol added to saline (Cat No. 1576708; USP). The EM model was created by administering a single dose of NTG at a concentration of 10 mg/kg, whereas the CM model was developed by administering the same concentration of NTG five times over 9 days on alternating days (Figure 1A).

### 4.3. Animals Were Divided into Four Groups Based on the Experimental Conditions

Episodic vehicle mouse model: mice received saline with propylene glycol (N = 15).

EM mouse model: mice received NTG (10 mg/kg) (N = 15).

Chronic vehicle mouse model: mice received saline with propylene glycol every other day, five times over 9 days (N = 15).

CM mouse model: mice received NTG (10 mg/kg) every other day, five times over 9 days (N = 15).

The episodic and chronic NTG-induced migraine models were distinguished by the duration and frequency of NTG injections. In the EM model, animals were euthanized 4 h after a single NTG injection, while in the CM model, animals were euthanized after receiving NTG injections five times over a span of 9 days.

### 4.4. Food Intake and Body Weight Measurement

Mice were acclimated for at least 1 week before being individually housed in a temperature-controlled room with a 12 h light/dark cycle (lights on at 8:00 a.m.). Body weight and food intake were monitored daily between 9:00 and 10:00 a.m. during the experimental period. All procedures were approved by the IACUC of Hallym University (Hallym #2022-80).

### 4.5. Tissue Collection

Mice were anesthetized with i.p. 2.5% avertin (2,2,2-Tribromoethanol (Cat No. T48402-25G; Sigma-Aldrich, St. Louis, MO, USA), and 2-Methyl-2-butanol (Cat No. 152463-250ML; Sigma-Aldrich, St. Louis, MO, USA)) and then perfused with 50 mL of phosphate-buffered saline (PBS; Cat No. PR2007-100-00; Biosesang, Seongnam, Republic of Korea) or saline through the left ventricle of the heart. The whole stomach, small intestine, and colon tissues were collected and fixed with 4% paraformaldehyde (PFA; Cat No. 30525-89-4; Sigma-Aldrich, St. Louis, MO, USA) for 16 h.

### 4.6. Immunohistochemical Staining

Stomach, small intestine, and colon tissue sections were deparaffinized in xylene and rehydrated through a graded alcohol series (100%, 90%, 80%, 70%, and 50%). For antigen retrieval (heat-induced epitope retrieval), samples were treated with citrate buffer (10 mM sodium citrate and 0.05% Tween 20, pH 6.0). The sections were treated with blocking solution at room temperature (RT) for 1 h, followed by incubation with the primary antibody against CGRP (Cat No. ab81887; 1:200, Abcam, Cambridge, UK) at 4 °C for 16 h. After washing three times, slides were incubated with the appropriate Alexa Fluor 488-conjugated secondary antibody (1:500) at RT for 1 h. For nuclear staining, slides were treated with DAPI (1:10,000) for 20 min before mounting. Images were obtained via fluorescence microscopy (Carl Zeiss 710; Carl Zeiss, Oberkochen, Germany), and the intensity of fluorescence was quantified using Image J (NIH, Bethesda, MD, USA) or Photoshop CS6 (Adobe Systems, San Jose, CA, USA).

### 4.7. cDNA Synthesis

Stomach (episodic vehicle, N = 5; episodic migraine, N = 5; chronic vehicle, N = 5; chronic migraine, N = 5), small intestine (duodenum and ileum) (episodic vehicle, N = 5; episodic migraine, N = 5; chronic vehicle, N = 5; chronic migraine, N = 5), and colon (proximal and distal regions) (episodic vehicle, N = 5; episodic migraine, N = 5; chronic vehicle, N = 5; chronic migraine, N = 5) tissue samples were collected in TRIzol reagent (Cat No.15596018; Invitrogen TRIzol™ Reagent, Invitrogen, CA, USA) and stored at −80 °C until processing. Total RNA was isolated from gut tissues, and cDNA was synthesized using Moloney murine leukemia virus reverse transcriptase (Invitrogen, Carlsbad, CA, USA). Reverse transcription polymerase chain reaction (RT-PCR) was performed with a cDNA template, primer, dNTP, 10× buffer, and Taq polymerase.

### 4.8. Real-Time Quantitative Reverse Transcription PCR

Real-time quantitative RT-PCR (qRT-PCR) was performed with SYBR Green (Cat No. K-6254; Bioneer AccuPower^®^ 2X GreenStar™ qPCR MasterMix (-ROX Dye), Daejeon, Republic of Korea) using a Bio-Rad real-time PCR detection system (Bio-Rad, Hercules, CA, USA). GAPDH was used as the internal control and confirmed to be stable across all tissues. The primers used were as follows:

IL-1β, 5′-CCTGTTGCTTAGCCGTAT-3′, 5′-ACTCTTCCACCTTCGATGC-3;

IL-6, 5′-CAGCTAGTTGTCATCCTGCT-3′, 5′-ACCTCGTTCAAAATGCCGATG-3′;

TNF-α, 5′-TTCGAGTGACAAGCCTGTAG-3′, 5′CTTTGAGATCCATGCCGTTG-3′;

IL-4, 5′-TTCACCATGGAATCCGTGTC-3′, 5′-GTCTTGGCCGAGGACTAAGG-3′;

IL-10, 5′-CTCTGATACCTCAGTTCCCA-3′, 5′-GTCCCCAATGGAAACAGCTT-3′;

TGF-β, 5′-TGCGCTTGCAGAGATTAAAA-3′, 5′-CTGCCGTACAACTCCAGTGA-3′;

GAPDH, 5′-CCTGTTGCTGTAGCCGTAT-3′, 5′-ACTCTTCCACCTTCGATGC-3′.

### 4.9. Cell Preparation

Colon tissues (episodic vehicle, N = 5; episodic migraine, N = 5; chronic vehicle, N = 5; chronic migraine, N = 5) were incubated in 0.1 M Dithiothreitol (Cat No. 10197777001; Roche) at 37 °C with gentle shaking to remove the epithelial cell layer, and tissue fragments retained on a 100 µm cell strainer (BD Falcon, Franklin Lakes, NJ, USA) were collected for digestion. The samples were digested using a gentleMACS™ Octo Dissociator with Heaters (Cat No. 130-096-427; Miltenyi Biotec, Bergisch Gladbach, Germany) with Liberase TL (Cat No. 05401020001; Roche, Basel, Switzerland), Liberase DH (Cat No. 05401054001; Roche), and DNase I (Cat No. 11284932001; Roche). After enzymatic digestion, the cell suspension was passed through a 100 µm cell strainer, and red blood cells were lysed using ACK lysis buffer (150 mM NH4Cl (Cat No. AC3001; Georgiachem, Smyrna, GA, USA), 1 M KHCO3 (Cat No. PB1020; Georgiachem, Smyrna, GA, USA), and 0.5 M EDTA (Cat No. ED2041; Georgiachem, Smyrna, GA, USA)). After washing with DMEM (Cat No. SH30243.01; HyClone, Logan, UT, USA) containing 5% FBS and 1% penicillin/streptomycin, cells were counted using a hemocytometer (Cat No. HSU-0650030, Marienfeld Superior, Lauda-Königshofen, Germany).

### 4.10. Flow Cytometric Analysis

For flow cytometric analysis, cells in single-cell suspension were plated in each well of round-bottomed 96-well plates containing PBS supplemented with 2% fetal bovine serum (FBS). Pooling was performed for groups with insufficient cell numbers to ensure adequate staining. The cells were stained with fluorochrome-conjugated antibodies for 20 min at 4 °C. The antibodies used for cell labeling were as follows: BV605 anti-CD45.2 (Cat. No. 109841, clone: 104; BioLegend, San Diego, CA, USA), BV421 anti-CD4 (Cat. No. 100544, clone: RM4-5; BioLegend), BV421 anti-CD11c (Cat. No. 117329, clone: N418; BioLegend), APC anti-PD-L1 (Cat. No. 124312, clone: 10F.9G2; BioLegend), PerCP-Cy5.5 anti-CD107a (Cat. No. 121626, clone: 1D4B; BioLegend), PE-Cy7 anti-CD25 (Cat. No. 25-0251-82, clone: PC61.5; eBioscience, San Diego, CA, USA), PE-Cy7 anti-CD8a (Cat. No. 25-0081-82, clone: 53-6.7; eBioscience), PerCP-Cy5.5 anti-CD8a (Cat. No. 45-0081-82, clone: 53-6.7; eBioscience), FITC anti-F4/80 (Cat. No. 11-4801-82, clone: BM8; eBioscience), PE anti-Foxp3 (Cat. No. 12-4771-80, clone: NRRF-30; eBioscience), PE-Cy7 anti-CD11b (Cat. No. 552850, clone: M1/70; BD Biosciences, Franklin Lakes, NJ, USA), APC anti-NK1.1 (Cat. No. 554420, clone: PK136; BD Biosciences), PerCP-Cy5.5 anti-Ly6G (Cat. No. 565797, clone: 1A8; BD Biosciences), Alexa Fluor 488 anti-Ki-67 (Cat. No. 561165, clone: B56; BD Biosciences), PE anti-NK1.1 (Cat. No. 553165, clone: PK136; BD Biosciences) FITC anti-IFN-γ (Cat. No. 554411, clone: XMG1.2; BD Biosciences), and APC anti-TNF (Cat. No. 554420, clone: MP6-XT22; BD Biosciences). A LIVE/DEAD Fixable Dead Cell Stain Kit (Invitrogen) was used to remove the dead cell population in all staining procedures. In addition, for intracellular staining, cells were first stained for surface antigens followed by permeabilization and fixation with either a Foxp3 Fixation/Permeabilization Kit (eBioscience) or Cytofix/Cytoperm Kit (BD Biosciences) according to the respective manufacturer’s instructions before addition of the appropriate antibodies. All stained samples were analyzed using a FACS CantoII instrument (BD Biosciences) and FlowJo software (version 10.10.0; Tree Star, Palo Alto, CA, USA).

### 4.11. Statistical Analysis

Data are expressed as the mean ± standard error of the mean (SEM). Statistical analyses were performed using GraphPad Prism (version 8; GraphPad Software, La Jolla, CA, USA), and group mean differences were evaluated via one-way analysis of variance (ANOVA). In all analyses, *p* < 0.05 was taken to indicate statistical significance.

## 5. Conclusions

In conclusion, this study provided preclinical evidence that migraine, especially CM, is associated with pronounced gut–brain axis dysregulation, particularly affecting the colon through GI inflammation, CGRP expression, immune cell alterations, and morphological changes. These results underscore the value of integrating GI and neurological perspectives in migraine research and support the development of gut-targeted interventions as potential disease-modifying strategies in migraine management.

## Data Availability

The original data presented in the study are openly available in FigShare at dx.doi.org/10.6084/m9.figshare.30226522.

## Supplementary Materials

The following supporting information can be downloaded at: https://www.mdpi.com/article/10.3390/ijms262110493/s1.

## Abbreviations

The following abbreviations are used in this manuscript:

NTG	Nitroglycerin;
EM	Episodic migraine;
CM	Chronic migraine;
CGRP	Calcitonin gene-related peptide;
GI	Gastrointestinal;
TNF-α	Tumor necrosis factor-alpha;
TGF-β	Transforming growth factor beta;
IL-1β	Interleukin-1β;
IL-6	Interleukin-6;
IL-8	Interleukin-8;
IL-4	Interleukin-4;
IL-10	Interleukin-10;
FACS	Fluorescence-activated cell sorting;
PFA	Paraformaldehyde;
Th1	Type 1 T helper cell;
Th17	Type 17 T helper cell;
Treg	Regulatory T cell.
