# Supplementary material for: Distinct Gut–Brain Axis Dysregulation in Episodic Versus Chronic Migraine: Insights from NTG-Induced Mouse Models
Source: Int J Mol Sci. 2025 Oct 29;26(21):10493. doi: 10.3390/ijms262110493 (PMC12607510; doi:10.3390/ijms262110493)
Supplement: Supplementary file 1 [file ijms-26-10493-s001.zip › Supplementary figures.pdf]

Figure		Type of Test	Group		N	Mean±SEM	P value
Figure 1B	Stomach	Parametric Test (One-way ANOVA with Tukey's multiple comparisons Test)	EM_VEH		5	1.08±0.037	P=0.4736
			EM_NTG		5	1.14±0.081	
			CM_VEH		5	1.1±0.032	
			CM_NTG		5	1.02±0.049	
	Small intestine	Parametric Test (One-way ANOVA with Tukey's multiple comparisons Test)	EM_VEH		5	29.44±0.857	P=0.3936
			EM_NTG		5	30.72±0.524	
			CM_VEH		5	29.6±0.805	
			CM_NTG		5	28.64±1.055	
	Colon	Parametric Test (One-way ANOVA with Tukey's multiple comparisons Test)	EM_VEH		5	9.90±0.17	***p=0.0003
			EM_NTG		5	9.60±0.292	
			CM_VEH		5	9.76±0.112	
			CM_NTG		5	8.3±0.255	
Figure 1c	Food intakes	Parametric Test (Two-way ANOVA with Sidak's multiple comparisons test)	VEH	1	5	0.00±0.00	Interaction ***p=0.0004
				2	5	5.93±0.445	
				3	5	12.49±0.185	
				4	5	18.35±0.269	
				5	5	25.09±0.246	
				6	5	31.47±0.286	
				7	5	36.83±0.306	Row factor ****P<0.0001
				8	5	42.78±0.237	
				9	5	49.08±0.224	
				10	5	54.13±0.324	
			NTG	1	5	0.00±0.00	
				2	5	3.93±0.268	
				3	5	9.04±0.112	
				4	5	15.36±0.547	
				5	5	21.84±0.524	Column factor ****P<0.0001
				6	5	27.87±0.587	
				7	5	33.52±0.485	
				8	5	39.28±0.457	
				9	5	45.54±0.677	
				10	5	50.88±0.712	
	Body weight	Parametric Test (Two-way ANOVA with Sidak's multiple comparisons test)	VEH	1	5	22.07±0.217	Interaction p=0.3195
				2	5	21.87±0.264	
				3	5	21.98±0.279	
				4	5	21.76±0.248	
				5	5	22.11±0.334	
				6	5	22.18±0.302	
				7	5	22.62±0.25	Row factor ****P<0.0001
				8	5	22.03±0.246	
				9	5	22.68±0.345	
				10	5	22.86±0.325	
			NTG	1	5	22.07±0.324	
				2	5	20.51±0.134	
				3	5	21.07±0.257	
				4	5	21.11±0.211	
				5	5	21.38±0.207	Column factor ****P<0.0001
				6	5	21.18±0.182	
				7	5	22.1±0.131	
				8	5	22.45±0.102	
				9	5	22.32±0.219	
				10	5	22.43±0.115	

Supplementary figure 1. Statistical analysis of gastrointestinal length measurements (stomach, small intestine, and colon) and changes in food intake and body weight in EM and CM models. Data are expressed as mean ± SEM (N=5 per group). Statistical analysis was performed using one-way or two-way ANOVA with Tukey's or Sidak's post-hoc tests, respectively.

Figure			Type of Test	Group	N	Mean±SEM	P value
Figure 2A	EM (Stomach)	*Il-1β*	Parametric Test (Unpaired T test)	VEH	5	1±0.295	p=0.2724
				NTG	5	1.5±0.3	
		Il-6	Parametric Test (Unpaired T test)	VEH	5	1±0.232	**p=0.0065
				NTG	5	2.2±0.233	
		Il-8	Parametric Test (Unpaired T test)	VEH	5	1±0.168	p=0.2656
				NTG	5	1.29±0.17	
		Tnfα	Parametric Test (Unpaired T test)	VEH	5	1±201	p=0.8884
				NTG	5	1.08±0.487	
		Il-4	Parametric Test (Unpaired T test)	VEH	5	1±0.263	p=0.7567
				NTG	5	1.1±0.187	
		Il-10	Parametric Test (Unpaired T test)	VEH	5	1±0.247	p=0.5182
				NTG	5	0.78±0.213	
		Tgfβ	Parametric Test (Unpaired T test)	VEH	5	1±0.352	p=0.5999
				NTG	5	1.48±0.803	
	CM (Stomach)	*Il-1β*	Parametric Test (Unpaired T test)	VEH	5	1±0.407	p=0.8432
				NTG	5	0.91±0.166	
		Il-6	Parametric Test (Unpaired T test)	VEH	5	1±0.259	p=0.099
				NTG	5	0.49±0.085	
		Il-8	Parametric Test (Unpaired T test)	VEH	5	1±0.3	p=0.0994
				NTG	5	0.42±0.084	
		Tnfα	Parametric Test (Unpaired T test)	VEH	5	1±0.242	p=0.0809
				NTG	5	0.49±0.085	
		Il-4	Parametric Test (Unpaired T test)	VEH	5	1±0.210	p=0.1094
				NTG	5	0.58±0.1	
		Il-10	Parametric Test (Unpaired T test)	VEH	5	1±0.327	p=0.108
				NTG	5	0.4±0.071	
		Tgfβ	Parametric Test (Unpaired T test)	VEH	5	1±0.257	*p=0.016
				NTG	5	2.41±0.385	

Supplementary figure 2. qRT-PCR analysis of cytokine expression (IL-1β, IL-6, IL-8, TNF-α, IL-4, IL-10, and TGF-β) in the stomach of EM and CM mice following NTG administration. Data represent mean ± SEM (N=5 per group). Statistical significance was determined using unpaired t-tests.

Figure			Type of Test	Group	N	Mean±SEM	P value
Figure 2B	EM (Duodenum)	*Il-1β*	Parametric Test (Unpaired T test)	VEH	5	1±0.185	*p=0.0162
				NTG	5	3.97±0.961	
		Il-6	Parametric Test (Unpaired T test)	VEH	5	1±0.262	****p<0.0001
				NTG	5	6.33±0.458	
		Il-8	Parametric Test (Unpaired T test)	VEH	5	1±0.192	p=0.081
				NTG	5	1.67±0.273	
		Tnfα	Parametric Test (Unpaired T test)	VEH	5	1±0.175	p=0.8333
				NTG	5	0.93±0.285	
		Il-4	Parametric Test (Unpaired T test)	VEH	5	1±0.112	*p=0.0488
				NTG	5	1.43±0.149	
		Il-10	Parametric Test (Unpaired T test)	VEH	5	1±0.227	p=0.0794
				NTG	5	1.59±0.188	
		Tgfβ	Parametric Test (Unpaired T test)	VEH	5	1±0.136	*p=0.046
				NTG	5	0.56±0.125	
	CM (Duodenum)	*Il-1β*	Parametric Test (Unpaired T test)	VEH	5	1±0.104	p=0.9424
				NTG	5	1.02±0.292	
		Il-6	Parametric Test (Unpaired T test)	VEH	5	1±0.256	p=0.8537
				NTG	5	0.94±0.218	
		Il-8	Parametric Test (Unpaired T test)	VEH	5	1±0.233	p=0.6455
				NTG	5	0.97±0.16	
		Tnfα	Parametric Test (Unpaired T test)	VEH	5	1±0.115	p=0.8953
				NTG	5	1.27±0.506	
		Il-4	Parametric Test (Unpaired T test)	VEH	5	1±0.12	p=0.672
				NTG	5	0.97±0.16	
		Il-10	Parametric Test (Unpaired T test)	VEH	5	1±0.156	p=0.547
				NTG	5	0.85±0.172	
		Tgfβ	Parametric Test (Unpaired T test)	VEH	5	1±0.201	p=0.31
				NTG	5	1.42±0.331	
	EM (Ileum)	*Il-1β*	Parametric Test (Unpaired T test)	VEH	5	1±0.2	p=0.3889
				NTG	5	0.68±0.283	
		Il-6	Parametric Test (Unpaired T test)	VEH	5	1±0.358	p=0.9925
				NTG	5	1±0.302	
		Il-8	Parametric Test (Unpaired T test)	VEH	5	1±0.223	p=0.4569
				NTG	5	1.32±0.346	
		Tnfα	Parametric Test (Unpaired T test)	VEH	5	1±0.18	p=0.6288
				NTG	5	1.31±0.593	
		Il-4	Parametric Test (Unpaired T test)	VEH	5	1±0.163	p=0.8822
				NTG	5	0.96±0.206	
		Il-10	Parametric Test (Unpaired T test)	VEH	5	1±0.244	p=0.4941
				NTG	5	1.26±0.276	
		Tgfβ	Parametric Test (Unpaired T test)	VEH	5	1±0.267	p=0.2129
				NTG	5	3.46±1.8	
	CM (Ileum)	*Il-1β*	Parametric Test (Unpaired T test)	VEH	5	1±0.287	p=0.5437
				NTG	5	0.81±0.085	
		Il-6	Parametric Test (Unpaired T test)	VEH	5	1±0.045	p=0.63
				NTG	5	0.8±0.404	
		Il-8	Parametric Test (Unpaired T test)	VEH	5	1±0.259	*p=0.0133
				NTG	5	0.16±0.056	
		Tnfα	Parametric Test (Unpaired T test)	VEH	5	1±0.145	***p=0.0007
				NTG	5	0.22±0.024	
		Il-4	Parametric Test (Unpaired T test)	VEH	5	1±0.355	*p=0.0455
				NTG	5	0.16±0.023	
		Il-10	Parametric Test (Unpaired T test)	VEH	5	1±0.192	p=0.066
				NTG	5	0.45±0.17	
		Tgfβ	Parametric Test (Unpaired T test)	VEH	5	1±0.665	p=0.2135
				NTG	5	2.09±0.451	

Supplementary figure 3. qRT-PCR analysis of cytokine expression in the duodenum and ileum of EM and CM mice. Cytokines analyzed include IL-1β, IL-6, IL-8, TNF-α, IL-4, IL-10, and TGF-β. Data are presented as mean ± SEM (N=5 per group). Parametric tests (unpaired t-tests) were used for comparisons.

Figure			Type of Test	Group	N	Mean±SEM	P value
Figure 2C	EM (Proximal colon)	*Il-1β*	Parametric Test (Unpaired T test)	VEH	5	1±0.4	p=0.1265
				NTG	5	2.85±1.005	
		Il-6	Parametric Test (Unpaired T test)	VEH	5	1±0.431	**p=0.0054
				NTG	5	4.07±0.69	
		Il-8	Parametric Test (Unpaired T test)	VEH	5	1±0.557	**p=0.0092
				NTG	5	5.40±1.162	
		Tnfα	Parametric Test (Unpaired T test)	VEH	5	1±0.701	p=0.0512
				NTG	5	55.68±23.863	
		Il-4	Parametric Test (Unpaired T test)	VEH	5	1±0.396	**p=0.0012
				NTG	5	5.75±0.893	
		Il-10	Parametric Test (Unpaired T test)	VEH	5	1±0.444	**p=0.0039
				NTG	5	4.84±0.848	
		Tgfβ	Parametric Test (Unpaired T test)	VEH	5	1±0.151	p=0.0671
				NTG	5	0.55±0.153	
	CM (Proximal colon)	*Il-1β*	Parametric Test (Unpaired T test)	VEH	5	1±0.163	p=0.1029
				NTG	5	1.74±0.369	
		Il-6	Parametric Test (Unpaired T test)	VEH	5	1±0.348	p=0.3653
				NTG	5	1.95±0.921	
		Il-8	Parametric Test (Unpaired T test)	VEH	5	1±0.443	p=0.9407
				NTG	5	1.05±0.42	
		Tnfα	Parametric Test (Unpaired T test)	VEH	5	1±0.291	p=0.7246
				NTG	5	1.13±0.228	
		Il-4	Parametric Test (Unpaired T test)	VEH	5	1±0.095	*p=0.0212
				NTG	5	1.51±0.151	
		Il-10	Parametric Test (Unpaired T test)	VEH	5	1±0.13	**p=0.0097
				NTG	5	2.3±0.362	
		Tgfβ	Parametric Test (Unpaired T test)	VEH	5	1±0.157	p=0.7882
				NTG	5	1.08±0.24	
	EM (Distal colon)	*Il-1β*	Parametric Test (Unpaired T test)	VEH	5	1±0.156	**p=0.0013
				NTG	5	5.66±0.946	
		Il-6	Parametric Test (Unpaired T test)	VEH	5	1±0.053	**p=0.0012
				NTG	5	1.55±0.099	
		Il-8	Parametric Test (Unpaired T test)	VEH	5	1±0.202	p=0.1653
				NTG	5	1.38±0.151	
		Tnfα	Parametric Test (Unpaired T test)	VEH	5	1±0.054	p=0.0937
				NTG	5	192.62±100.767	
		Il-4	Parametric Test (Unpaired T test)	VEH	5	1±0.28	**p=0.0091
				NTG	5	2.31±0.263	
		Il-10	Parametric Test (Unpaired T test)	VEH	5	1±0.241	p=0.8488
				NTG	5	1.06±0.204	
		Tgfβ	Parametric Test (Unpaired T test)	VEH	5	1±0.32	p=0.0661
				NTG	5	3.11±0.938	
	CM (Distal colon)	*Il-1β*	Parametric Test (Unpaired T test)	VEH	5	1±0.194	***p=0.0007
				NTG	5	2.77±0.273	
		Il-6	Parametric Test (Unpaired T test)	VEH	5	1±0.166	p=0.4507
				NTG	5	0.83±0.134	
		Il-8	Parametric Test (Unpaired T test)	VEH	5	1±0.199	p=0.0698
				NTG	5	0.51±0.12	
		Tnfα	Parametric Test (Unpaired T test)	VEH	5	1±0.223	p=0.3675
				NTG	5	0.73±0.171	
		Il-4	Parametric Test (Unpaired T test)	VEH	5	1±0.097	**p=0.0071
				NTG	5	0.51±0.097	
		Il-10	Parametric Test (Unpaired T test)	VEH	5	1±0.311	p=0.2536
				NTG	5	1.46±0.211	
		Tgfβ	Parametric Test (Unpaired T test)	VEH	5	1±0.314	*p=0.031
				NTG	5	5.78±1.803	

Supplementary figure 4. qRT-PCR analysis of cytokine expression in the proximal and distal colon of EM and CM mice. Expression patterns reveal site-specific inflammatory and anti-inflammatory responses. Data are expressed as mean ± SEM (N=5 per group). Statistical significance was assessed by unpaired t-test.

Figure		Type of Test	Group	N	Mean±SEM	P value
Figure 3A	Stomach	Parametric Test (One-way ANOVA with Tukey’s multiple comparisons Test)	EM_VEH	5	207.6±30.634	***0.0001
			EM_NTG	5	352±40.658	
			CM_VEH	5	240.4±32.571	
			CM_NTG	5	570±66.964	
Figure 3B	Small intestine	Parametric Test (One-way ANOVA with Tukey’s multiple comparisons Test)	EM_VEH	5	611.6±93.508	**0.0046
			EM_NTG	5	859.8±100.925	
			CM_VEH	5	714.4±91.861	
			CM_NTG	5	1390.2±216.695	
Figure 3C	Colon	Parametric Test (One-way ANOVA with Tukey’s multiple comparisons Test)	EM_VEH	5	423.2±96.881	****p<0.0001
			EM_NTG	5	1201.4±142.46	
			CM_VEH	3	480.4±49.774	
			CM_NTG	3	2122.8±305.473	

Supplementary figure 5. Quantitative analysis of CGRP fluorescence intensity in the stomach, small intestine, and colon of EM and CM models. Data are shown as mean ± SEM (N=5 per group). Statistical comparisons were made using one-way ANOVA with Tukey’s multiple-comparison test.

Figure		Type of Test	Group	N	Mean±SEM	P value
Figure 4A	T cells	Parametric Test (Unpaired T test)	VEH	4	6.87±0.688	0.0756
			NTG	5	5.42±0.296	
	B cells	Parametric Test (Unpaired T test)	VEH	5	4.96±0.666	0.9963
			NTG	5	4.96±0.507	
	Tregs	Parametric Test (Unpaired T test)	VEH	5	43.48±2.417	0.7232
			NTG	5	45.12±3.76	
	Th1 cells	Parametric Test (Unpaired T test)	VEH	4	3.66±0.59	0.8427
			NTG	5	3.45±0.767	
	Th17 cells	Parametric Test (Unpaired T test)	VEH	5	47.14±2.689	**0.0085
			NTG	5	60.66±2.828	
	Macrophages	Parametric Test (Unpaired T test)	VEH	5	19.42±1.091	0.0687
			NTG	4	24.13±2.057	
Figure 4B	T cells	Parametric Test (Unpaired T test)	VEH	5	4.52±0.421	0.0897
			NTG	5	3.57±0.254	
	B cells	Parametric Test (Unpaired T test)	VEH	5	42.08±3.142	*0.0199
			NTG	5	27.16±4.072	
	Tregs	Parametric Test (Unpaired T test)	VEH	5	32.22±4.758	0.2349
			NTG	5	40.1±3.873	
	Th1 cells	Parametric Test (Unpaired T test)	VEH	5	2.2±0.211	0.8306
			NTG	5	2.65±0.654	
	Th17 cells	Parametric Test (Unpaired T test)	VEH	5	36.28±1.607	*0.0189
			NTG	4	49.5±4.496	
	Macrophages	Parametric Test (Unpaired T test)	VEH	4	12.73±0.437	0.1752
			NTG	3	16.9±3.1	
Figure 5A	T cells	Parametric Test (Unpaired T test)	VEH	5	10.92±0.445	0.675
			NTG	4	10.6±0.611	
	B cells	Parametric Test (Unpaired T test)	VEH	5	6.53±5.62	0.4243
			NTG	4	5.62±0.277	
	Tregs	Parametric Test (Unpaired T test)	VEH	5	27.16±2.19	0.0788
			NTG	5	22.18±1.149	
	Th1 cells	Parametric Test (Unpaired T test)	VEH	5	5.21±0.434	0.5164
			NTG	5	4.72±0.592	
	Th17 cells	Parametric Test (Unpaired T test)	VEH	5	55.16±0.95	0.8524
			NTG	5	54.4±3.839	
	Macrophages	Parametric Test (Unpaired T test)	VEH	4	19.05±0.484	0.6749
			NTG	4	19.73±1.453	
Figure 5B	T cells	Parametric Test (Unpaired T test)	VEH	4	7.02±0.41	*0.0221
			NTG	4	9.55±0.715	
	B cells	Parametric Test (Unpaired T test)	VEH	4	50.03±3.147	*0.0131
			NTG	5	38.34±1.948	
	Tregs	Parametric Test (Unpaired T test)	VEH	4	11.79±0.872	*0.0114
			NTG	5	21.82±2.512	
	Th1 cells	Parametric Test (Unpaired T test)	VEH	5	4.45±0.511	0.0932
			NTG	5	3.17±0.441	
	Th17 cells	Parametric Test (Unpaired T test)	VEH	5	39.56±5.224	0.4271
			NTG	5	34±4.11	
	Macrophages	Parametric Test (Unpaired T test)	VEH	4	11.18±1.465	**0.0062
			NTG	5	18.36±1.186	

Supplementary figure 6. Flow cytometric analysis was conducted to quantify immune cell populations in the colonic tissues of EM and CM mice, including T cells, B cells, regulatory T cells (Tregs), Th1 cells, Th17 cells, and macrophages. Data are presented as mean ± SEM (n = 3–5 per group). Statistical analysis was performed using unpaired t-tests to compare vehicle- and NTG-treated groups within each experimental condition.
