# Supplementary material for: Distinct Gut–Brain Axis Dysregulation in Episodic Versus Chronic Migraine: Insights from NTG-Induced Mouse Models
Source: Int J Mol Sci. 2025 Oct 29;26(21):10493. doi: 10.3390/ijms262110493 (PMC12607510; doi:10.3390/ijms262110493)
Supplement: Supplementary file 1 [file ijms-26-10493-s001.zip › Supplementary figures.pptx]

## Slide 1
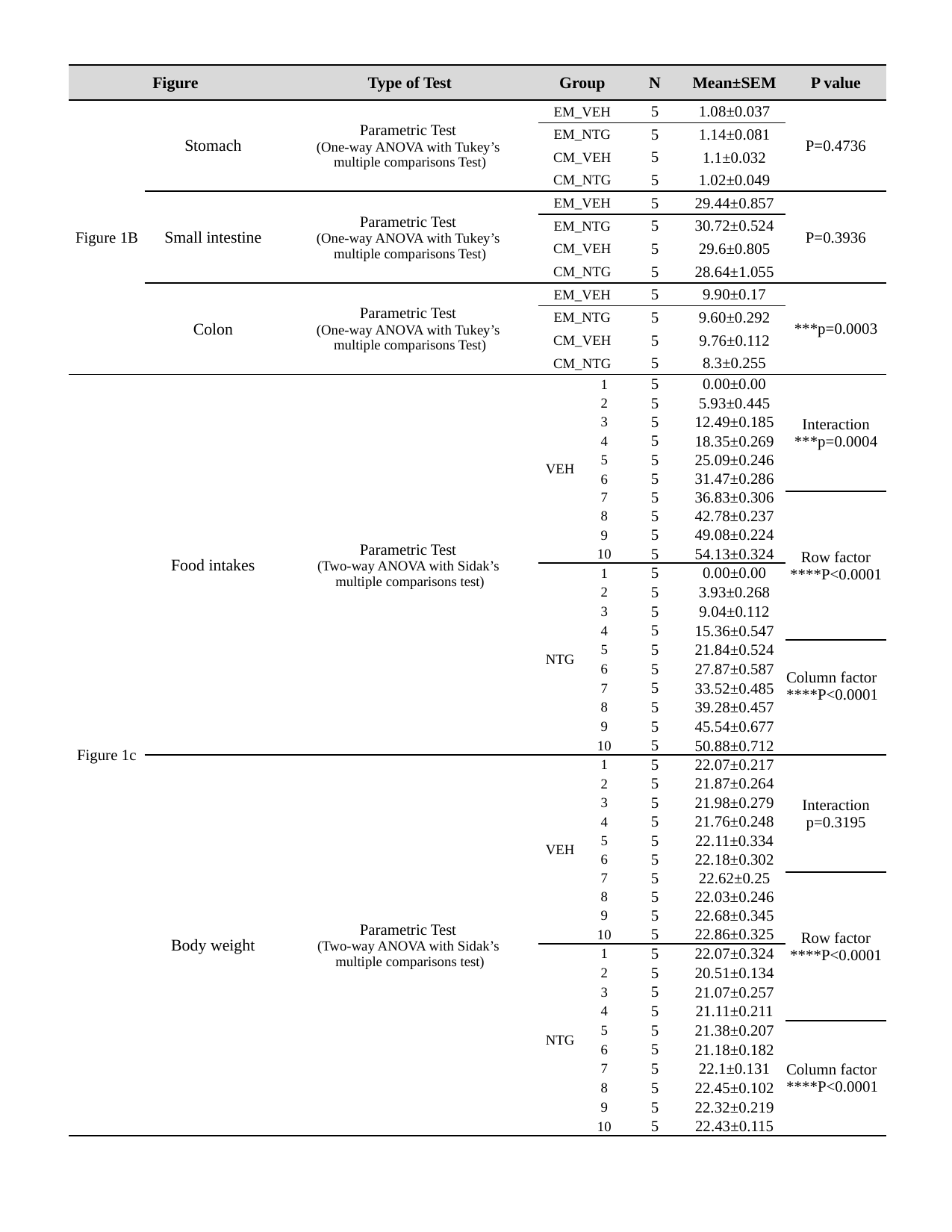

| Figure | | Type of Test | Group | | N | Mean±SEM | P value |
| --- | --- | --- | --- | --- | --- | --- | --- |
| Figure 1B | Stomach | Parametric Test (One-way ANOVA with Tukey’s multiple comparisons Test) | EM\_VEH | | 5 | 1.08±0.037 | P=0.4736 |
| | | | EM\_NTG | | 5 | 1.14±0.081 | |
| | | | CM\_VEH | | 5 | 1.1±0.032 | |
| | | | CM\_NTG | | 5 | 1.02±0.049 | |
| | Small intestine | Parametric Test (One-way ANOVA with Tukey’s multiple comparisons Test) | EM\_VEH | | 5 | 29.44±0.857 | P=0.3936 |
| | | | EM\_NTG | | 5 | 30.72±0.524 | |
| | | | CM\_VEH | | 5 | 29.6±0.805 | |
| | | | CM\_NTG | | 5 | 28.64±1.055 | |
| | Colon | Parametric Test (One-way ANOVA with Tukey’s multiple comparisons Test) | EM\_VEH | | 5 | 9.90±0.17 | \*\*\*p=0.0003 |
| | | | EM\_NTG | | 5 | 9.60±0.292 | |
| | | | CM\_VEH | | 5 | 9.76±0.112 | |
| | | | CM\_NTG | | 5 | 8.3±0.255 | |
| Figure 1c | Food intakes | Parametric Test (Two-way ANOVA with Sidak’s multiple comparisons test) | VEH | 1 | 5 | 0.00±0.00 | Interaction \*\*\*p=0.0004 |
| | | | | 2 | 5 | 5.93±0.445 | |
| | | | | 3 | 5 | 12.49±0.185 | |
| | | | | 4 | 5 | 18.35±0.269 | |
| | | | | 5 | 5 | 25.09±0.246 | |
| | | | | 6 | 5 | 31.47±0.286 | |
| | | | | 7 | 5 | 36.83±0.306 | |
| | | | | | | | Row factor \*\*\*\*P<0.0001 |
| | | | | 8 | 5 | 42.78±0.237 | |
| | | | | 9 | 5 | 49.08±0.224 | |
| | | | | 10 | 5 | 54.13±0.324 | |
| | | | NTG | 1 | 5 | 0.00±0.00 | |
| | | | | 2 | 5 | 3.93±0.268 | |
| | | | | 3 | 5 | 9.04±0.112 | |
| | | | | 4 | 5 | 15.36±0.547 | |
| | | | | 5 | 5 | 21.84±0.524 | Column factor \*\*\*\*P<0.0001 |
| | | | | 6 | 5 | 27.87±0.587 | |
| | | | | 7 | 5 | 33.52±0.485 | |
| | | | | 8 | 5 | 39.28±0.457 | |
| | | | | 9 | 5 | 45.54±0.677 | |
| | | | | 10 | 5 | 50.88±0.712 | |
| | Body weight | Parametric Test (Two-way ANOVA with Sidak’s multiple comparisons test) | VEH | 1 | 5 | 22.07±0.217 | Interaction p=0.3195 |
| | | | | 2 | 5 | 21.87±0.264 | |
| | | | | 3 | 5 | 21.98±0.279 | |
| | | | | 4 | 5 | 21.76±0.248 | |
| | | | | 5 | 5 | 22.11±0.334 | |
| | | | | 6 | 5 | 22.18±0.302 | |
| | | | | 7 | 5 | 22.62±0.25 | |
| | | | | | | | Row factor \*\*\*\*P<0.0001 |
| | | | | 8 | 5 | 22.03±0.246 | |
| | | | | 9 | 5 | 22.68±0.345 | |
| | | | | 10 | 5 | 22.86±0.325 | |
| | | | NTG | 1 | 5 | 22.07±0.324 | |
| | | | | 2 | 5 | 20.51±0.134 | |
| | | | | 3 | 5 | 21.07±0.257 | |
| | | | | 4 | 5 | 21.11±0.211 | |
| | | | | 5 | 5 | 21.38±0.207 | Column factor \*\*\*\*P<0.0001 |
| | | | | 6 | 5 | 21.18±0.182 | |
| | | | | 7 | 5 | 22.1±0.131 | |
| | | | | 8 | 5 | 22.45±0.102 | |
| | | | | 9 | 5 | 22.32±0.219 | |
| | | | | 10 | 5 | 22.43±0.115 | |

## Slide 2
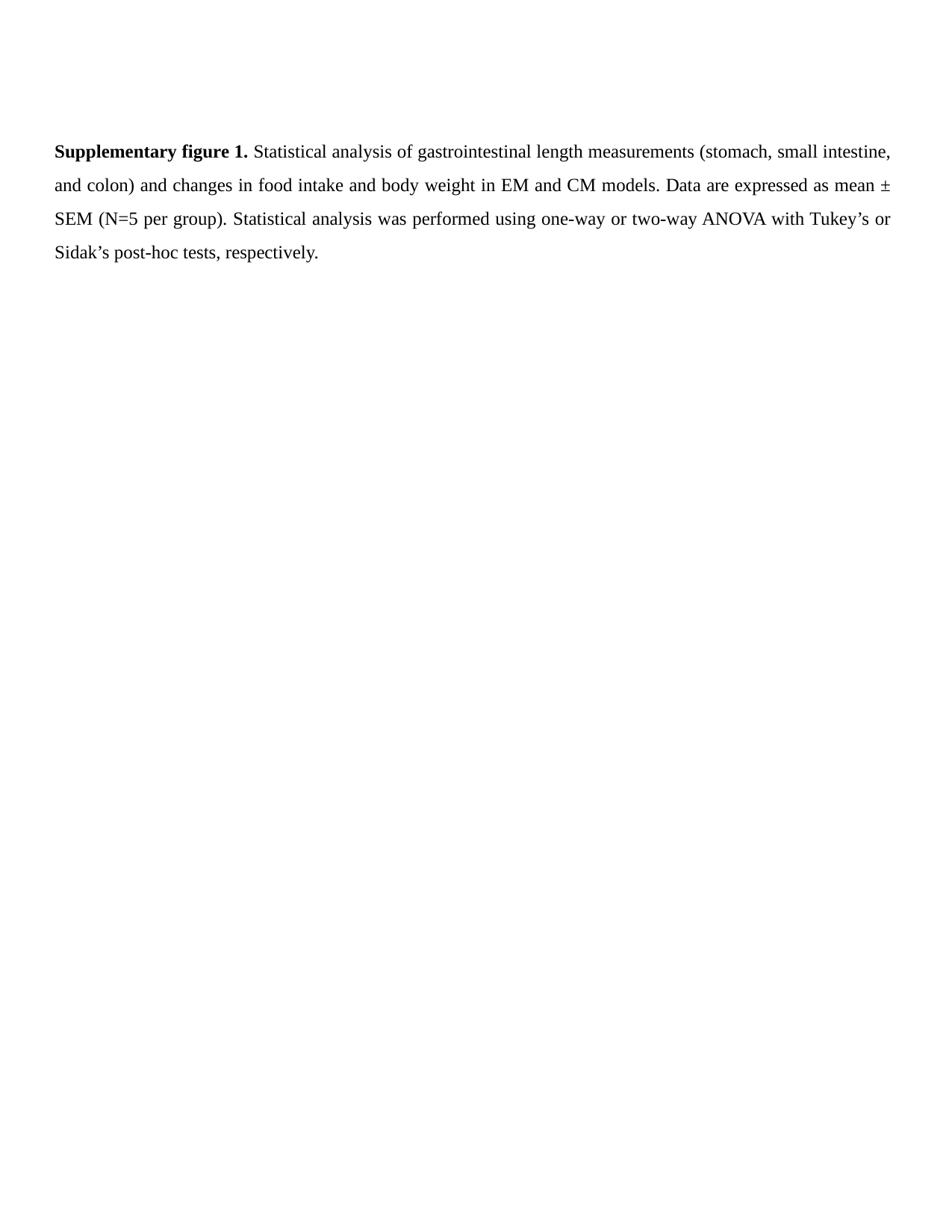

Supplementary figure 1. Statistical analysis of gastrointestinal length measurements (stomach, small intestine, and colon) and changes in food intake and body weight in EM and CM models. Data are expressed as mean ± SEM (N=5 per group). Statistical analysis was performed using one-way or two-way ANOVA with Tukey’s or Sidak’s post-hoc tests, respectively.

## Slide 3
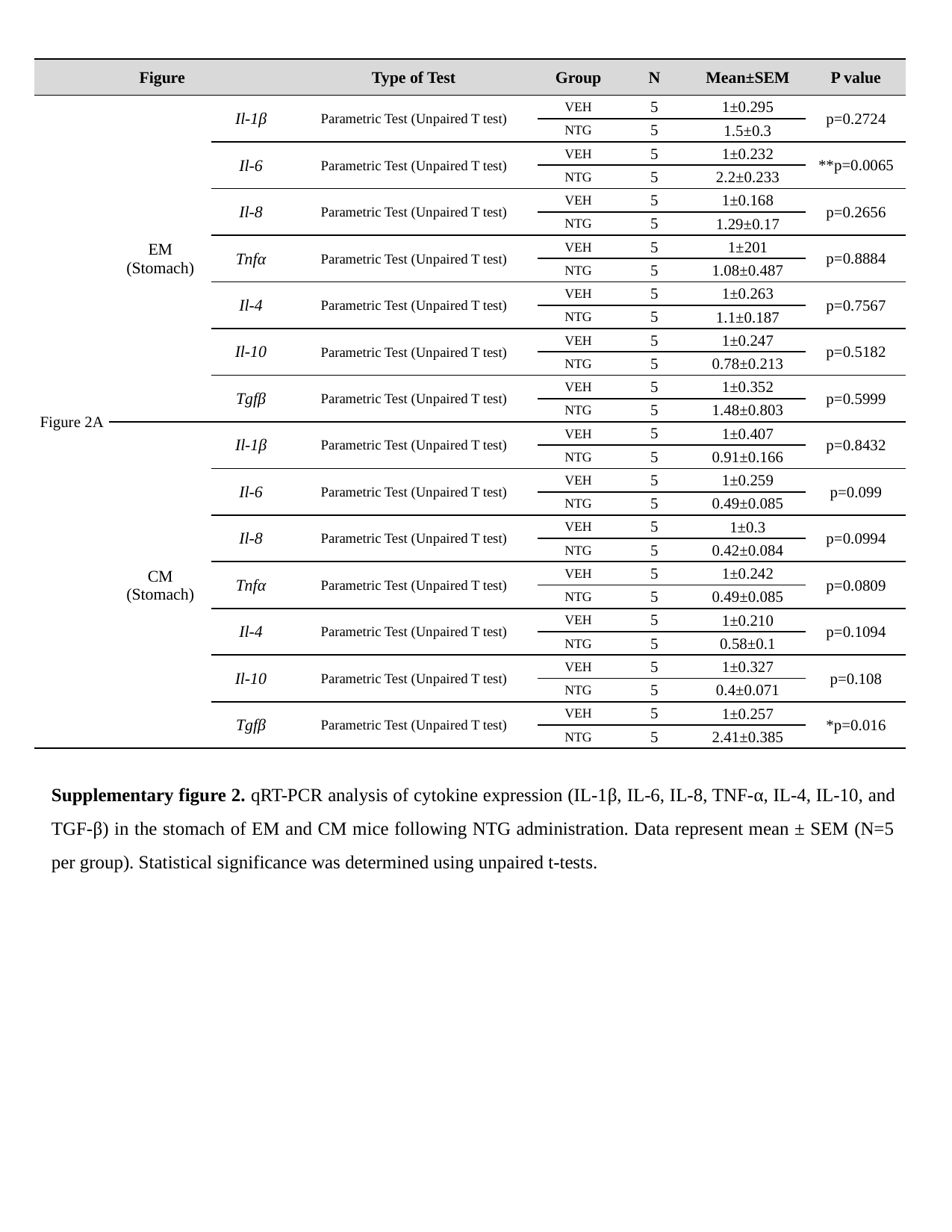

| Figure | | | Type of Test | Group | N | Mean±SEM | P value |
| --- | --- | --- | --- | --- | --- | --- | --- |
| Figure 2A | EM (Stomach) | Il-1β | Parametric Test (Unpaired T test) | VEH | 5 | 1±0.295 | p=0.2724 |
| | | | | NTG | 5 | 1.5±0.3 | |
| | | Il-6 | Parametric Test (Unpaired T test) | VEH | 5 | 1±0.232 | \*\*p=0.0065 |
| | | | | NTG | 5 | 2.2±0.233 | |
| | | Il-8 | Parametric Test (Unpaired T test) | VEH | 5 | 1±0.168 | p=0.2656 |
| | | | | NTG | 5 | 1.29±0.17 | |
| | | Tnfα | Parametric Test (Unpaired T test) | VEH | 5 | 1±201 | p=0.8884 |
| | | | | NTG | 5 | 1.08±0.487 | |
| | | Il-4 | Parametric Test (Unpaired T test) | VEH | 5 | 1±0.263 | p=0.7567 |
| | | | | NTG | 5 | 1.1±0.187 | |
| | | Il-10 | Parametric Test (Unpaired T test) | VEH | 5 | 1±0.247 | p=0.5182 |
| | | | | NTG | 5 | 0.78±0.213 | |
| | | Tgfβ | Parametric Test (Unpaired T test) | VEH | 5 | 1±0.352 | p=0.5999 |
| | | | | NTG | 5 | 1.48±0.803 | |
| | CM (Stomach) | Il-1β | Parametric Test (Unpaired T test) | VEH | 5 | 1±0.407 | p=0.8432 |
| | | | | NTG | 5 | 0.91±0.166 | |
| | | Il-6 | Parametric Test (Unpaired T test) | VEH | 5 | 1±0.259 | p=0.099 |
| | | | | NTG | 5 | 0.49±0.085 | |
| | | Il-8 | Parametric Test (Unpaired T test) | VEH | 5 | 1±0.3 | p=0.0994 |
| | | | | NTG | 5 | 0.42±0.084 | |
| | | Tnfα | Parametric Test (Unpaired T test) | VEH | 5 | 1±0.242 | p=0.0809 |
| | | | | NTG | 5 | 0.49±0.085 | |
| | | Il-4 | Parametric Test (Unpaired T test) | VEH | 5 | 1±0.210 | p=0.1094 |
| | | | | NTG | 5 | 0.58±0.1 | |
| | | Il-10 | Parametric Test (Unpaired T test) | VEH | 5 | 1±0.327 | p=0.108 |
| | | | | NTG | 5 | 0.4±0.071 | |
| | | Tgfβ | Parametric Test (Unpaired T test) | VEH | 5 | 1±0.257 | \*p=0.016 |
| | | | | NTG | 5 | 2.41±0.385 | |
Supplementary figure 2. qRT-PCR analysis of cytokine expression (IL-1β, IL-6, IL-8, TNF-α, IL-4, IL-10, and TGF-β) in the stomach of EM and CM mice following NTG administration. Data represent mean ± SEM (N=5 per group). Statistical significance was determined using unpaired t-tests.

## Slide 4
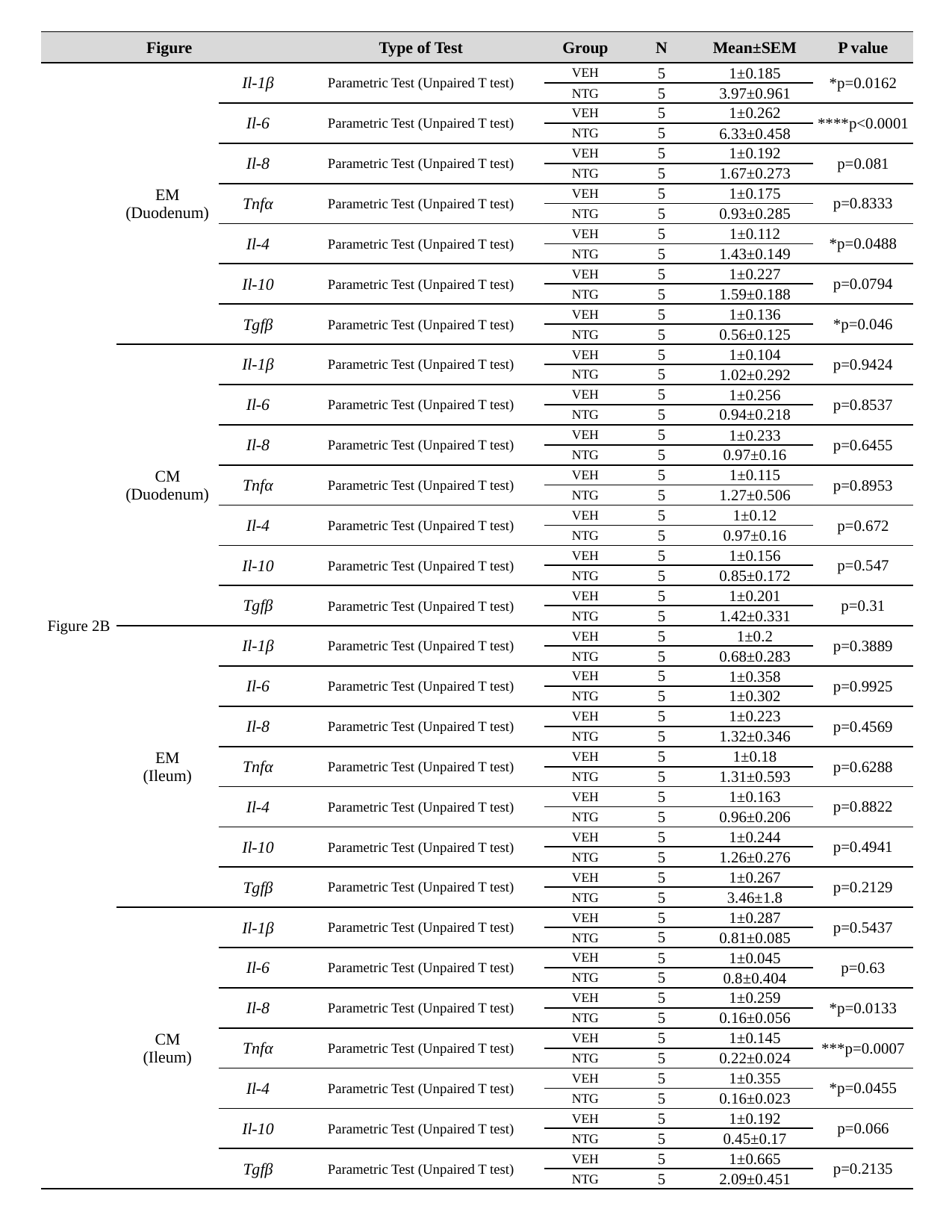

| Figure | | | Type of Test | Group | N | Mean±SEM | P value |
| --- | --- | --- | --- | --- | --- | --- | --- |
| Figure 2B | EM (Duodenum) | Il-1β | Parametric Test (Unpaired T test) | VEH | 5 | 1±0.185 | \*p=0.0162 |
| | | | | NTG | 5 | 3.97±0.961 | |
| | | Il-6 | Parametric Test (Unpaired T test) | VEH | 5 | 1±0.262 | \*\*\*\*p<0.0001 |
| | | | | NTG | 5 | 6.33±0.458 | |
| | | Il-8 | Parametric Test (Unpaired T test) | VEH | 5 | 1±0.192 | p=0.081 |
| | | | | NTG | 5 | 1.67±0.273 | |
| | | Tnfα | Parametric Test (Unpaired T test) | VEH | 5 | 1±0.175 | p=0.8333 |
| | | | | NTG | 5 | 0.93±0.285 | |
| | | Il-4 | Parametric Test (Unpaired T test) | VEH | 5 | 1±0.112 | \*p=0.0488 |
| | | | | NTG | 5 | 1.43±0.149 | |
| | | Il-10 | Parametric Test (Unpaired T test) | VEH | 5 | 1±0.227 | p=0.0794 |
| | | | | NTG | 5 | 1.59±0.188 | |
| | | Tgfβ | Parametric Test (Unpaired T test) | VEH | 5 | 1±0.136 | \*p=0.046 |
| | | | | NTG | 5 | 0.56±0.125 | |
| | CM (Duodenum) | Il-1β | Parametric Test (Unpaired T test) | VEH | 5 | 1±0.104 | p=0.9424 |
| | | | | NTG | 5 | 1.02±0.292 | |
| | | Il-6 | Parametric Test (Unpaired T test) | VEH | 5 | 1±0.256 | p=0.8537 |
| | | | | NTG | 5 | 0.94±0.218 | |
| | | Il-8 | Parametric Test (Unpaired T test) | VEH | 5 | 1±0.233 | p=0.6455 |
| | | | | NTG | 5 | 0.97±0.16 | |
| | | Tnfα | Parametric Test (Unpaired T test) | VEH | 5 | 1±0.115 | p=0.8953 |
| | | | | NTG | 5 | 1.27±0.506 | |
| | | Il-4 | Parametric Test (Unpaired T test) | VEH | 5 | 1±0.12 | p=0.672 |
| | | | | NTG | 5 | 0.97±0.16 | |
| | | Il-10 | Parametric Test (Unpaired T test) | VEH | 5 | 1±0.156 | p=0.547 |
| | | | | NTG | 5 | 0.85±0.172 | |
| | | Tgfβ | Parametric Test (Unpaired T test) | VEH | 5 | 1±0.201 | p=0.31 |
| | | | | NTG | 5 | 1.42±0.331 | |
| | EM (Ileum) | Il-1β | Parametric Test (Unpaired T test) | VEH | 5 | 1±0.2 | p=0.3889 |
| | | | | NTG | 5 | 0.68±0.283 | |
| | | Il-6 | Parametric Test (Unpaired T test) | VEH | 5 | 1±0.358 | p=0.9925 |
| | | | | NTG | 5 | 1±0.302 | |
| | | Il-8 | Parametric Test (Unpaired T test) | VEH | 5 | 1±0.223 | p=0.4569 |
| | | | | NTG | 5 | 1.32±0.346 | |
| | | Tnfα | Parametric Test (Unpaired T test) | VEH | 5 | 1±0.18 | p=0.6288 |
| | | | | NTG | 5 | 1.31±0.593 | |
| | | Il-4 | Parametric Test (Unpaired T test) | VEH | 5 | 1±0.163 | p=0.8822 |
| | | | | NTG | 5 | 0.96±0.206 | |
| | | Il-10 | Parametric Test (Unpaired T test) | VEH | 5 | 1±0.244 | p=0.4941 |
| | | | | NTG | 5 | 1.26±0.276 | |
| | | Tgfβ | Parametric Test (Unpaired T test) | VEH | 5 | 1±0.267 | p=0.2129 |
| | | | | NTG | 5 | 3.46±1.8 | |
| | CM (Ileum) | Il-1β | Parametric Test (Unpaired T test) | VEH | 5 | 1±0.287 | p=0.5437 |
| | | | | NTG | 5 | 0.81±0.085 | |
| | | Il-6 | Parametric Test (Unpaired T test) | VEH | 5 | 1±0.045 | p=0.63 |
| | | | | NTG | 5 | 0.8±0.404 | |
| | | Il-8 | Parametric Test (Unpaired T test) | VEH | 5 | 1±0.259 | \*p=0.0133 |
| | | | | NTG | 5 | 0.16±0.056 | |
| | | Tnfα | Parametric Test (Unpaired T test) | VEH | 5 | 1±0.145 | \*\*\*p=0.0007 |
| | | | | NTG | 5 | 0.22±0.024 | |
| | | Il-4 | Parametric Test (Unpaired T test) | VEH | 5 | 1±0.355 | \*p=0.0455 |
| | | | | NTG | 5 | 0.16±0.023 | |
| | | Il-10 | Parametric Test (Unpaired T test) | VEH | 5 | 1±0.192 | p=0.066 |
| | | | | NTG | 5 | 0.45±0.17 | |
| | | Tgfβ | Parametric Test (Unpaired T test) | VEH | 5 | 1±0.665 | p=0.2135 |
| | | | | NTG | 5 | 2.09±0.451 | |

## Slide 5
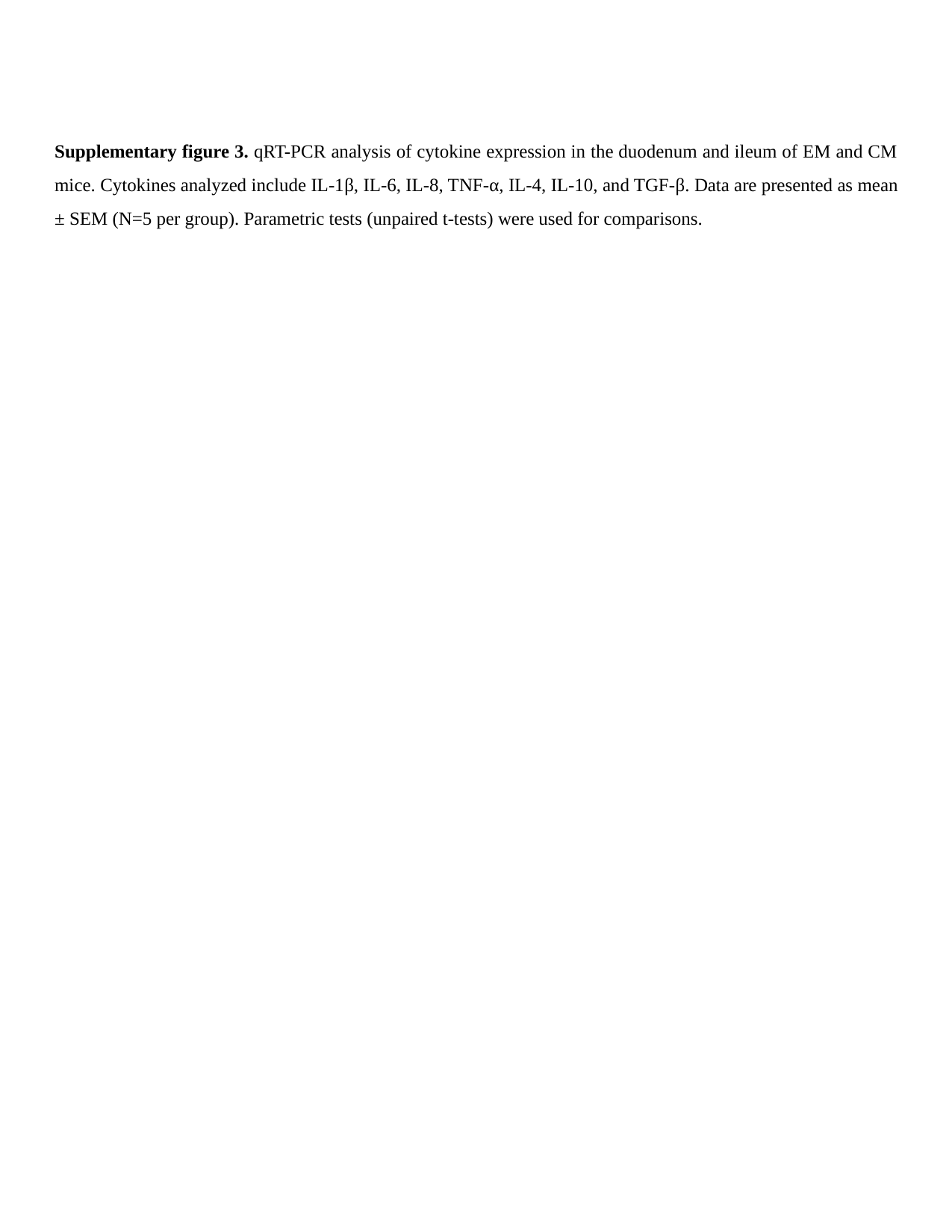

Supplementary figure 3. qRT-PCR analysis of cytokine expression in the duodenum and ileum of EM and CM mice. Cytokines analyzed include IL-1β, IL-6, IL-8, TNF-α, IL-4, IL-10, and TGF-β. Data are presented as mean ± SEM (N=5 per group). Parametric tests (unpaired t-tests) were used for comparisons.

## Slide 6
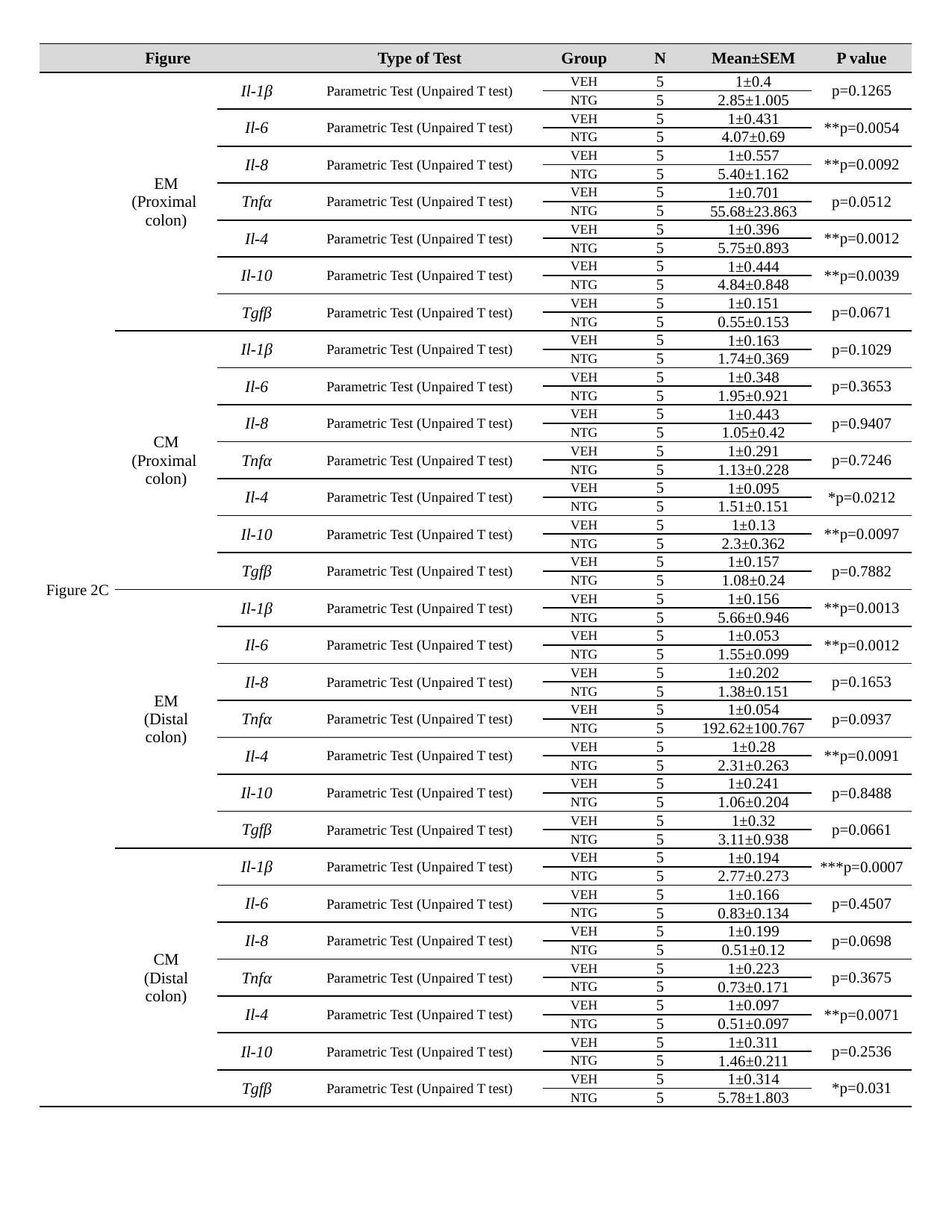

| Figure | | | Type of Test | Group | N | Mean±SEM | P value |
| --- | --- | --- | --- | --- | --- | --- | --- |
| Figure 2C | EM (Proximal colon) | Il-1β | Parametric Test (Unpaired T test) | VEH | 5 | 1±0.4 | p=0.1265 |
| | | | | NTG | 5 | 2.85±1.005 | |
| | | Il-6 | Parametric Test (Unpaired T test) | VEH | 5 | 1±0.431 | \*\*p=0.0054 |
| | | | | NTG | 5 | 4.07±0.69 | |
| | | Il-8 | Parametric Test (Unpaired T test) | VEH | 5 | 1±0.557 | \*\*p=0.0092 |
| | | | | NTG | 5 | 5.40±1.162 | |
| | | Tnfα | Parametric Test (Unpaired T test) | VEH | 5 | 1±0.701 | p=0.0512 |
| | | | | NTG | 5 | 55.68±23.863 | |
| | | Il-4 | Parametric Test (Unpaired T test) | VEH | 5 | 1±0.396 | \*\*p=0.0012 |
| | | | | NTG | 5 | 5.75±0.893 | |
| | | Il-10 | Parametric Test (Unpaired T test) | VEH | 5 | 1±0.444 | \*\*p=0.0039 |
| | | | | NTG | 5 | 4.84±0.848 | |
| | | Tgfβ | Parametric Test (Unpaired T test) | VEH | 5 | 1±0.151 | p=0.0671 |
| | | | | NTG | 5 | 0.55±0.153 | |
| | CM (Proximal colon) | Il-1β | Parametric Test (Unpaired T test) | VEH | 5 | 1±0.163 | p=0.1029 |
| | | | | NTG | 5 | 1.74±0.369 | |
| | | Il-6 | Parametric Test (Unpaired T test) | VEH | 5 | 1±0.348 | p=0.3653 |
| | | | | NTG | 5 | 1.95±0.921 | |
| | | Il-8 | Parametric Test (Unpaired T test) | VEH | 5 | 1±0.443 | p=0.9407 |
| | | | | NTG | 5 | 1.05±0.42 | |
| | | Tnfα | Parametric Test (Unpaired T test) | VEH | 5 | 1±0.291 | p=0.7246 |
| | | | | NTG | 5 | 1.13±0.228 | |
| | | Il-4 | Parametric Test (Unpaired T test) | VEH | 5 | 1±0.095 | \*p=0.0212 |
| | | | | NTG | 5 | 1.51±0.151 | |
| | | Il-10 | Parametric Test (Unpaired T test) | VEH | 5 | 1±0.13 | \*\*p=0.0097 |
| | | | | NTG | 5 | 2.3±0.362 | |
| | | Tgfβ | Parametric Test (Unpaired T test) | VEH | 5 | 1±0.157 | p=0.7882 |
| | | | | NTG | 5 | 1.08±0.24 | |
| | EM (Distal colon) | Il-1β | Parametric Test (Unpaired T test) | VEH | 5 | 1±0.156 | \*\*p=0.0013 |
| | | | | NTG | 5 | 5.66±0.946 | |
| | | Il-6 | Parametric Test (Unpaired T test) | VEH | 5 | 1±0.053 | \*\*p=0.0012 |
| | | | | NTG | 5 | 1.55±0.099 | |
| | | Il-8 | Parametric Test (Unpaired T test) | VEH | 5 | 1±0.202 | p=0.1653 |
| | | | | NTG | 5 | 1.38±0.151 | |
| | | Tnfα | Parametric Test (Unpaired T test) | VEH | 5 | 1±0.054 | p=0.0937 |
| | | | | NTG | 5 | 192.62±100.767 | |
| | | Il-4 | Parametric Test (Unpaired T test) | VEH | 5 | 1±0.28 | \*\*p=0.0091 |
| | | | | NTG | 5 | 2.31±0.263 | |
| | | Il-10 | Parametric Test (Unpaired T test) | VEH | 5 | 1±0.241 | p=0.8488 |
| | | | | NTG | 5 | 1.06±0.204 | |
| | | Tgfβ | Parametric Test (Unpaired T test) | VEH | 5 | 1±0.32 | p=0.0661 |
| | | | | NTG | 5 | 3.11±0.938 | |
| | CM (Distal colon) | Il-1β | Parametric Test (Unpaired T test) | VEH | 5 | 1±0.194 | \*\*\*p=0.0007 |
| | | | | NTG | 5 | 2.77±0.273 | |
| | | Il-6 | Parametric Test (Unpaired T test) | VEH | 5 | 1±0.166 | p=0.4507 |
| | | | | NTG | 5 | 0.83±0.134 | |
| | | Il-8 | Parametric Test (Unpaired T test) | VEH | 5 | 1±0.199 | p=0.0698 |
| | | | | NTG | 5 | 0.51±0.12 | |
| | | Tnfα | Parametric Test (Unpaired T test) | VEH | 5 | 1±0.223 | p=0.3675 |
| | | | | NTG | 5 | 0.73±0.171 | |
| | | Il-4 | Parametric Test (Unpaired T test) | VEH | 5 | 1±0.097 | \*\*p=0.0071 |
| | | | | NTG | 5 | 0.51±0.097 | |
| | | Il-10 | Parametric Test (Unpaired T test) | VEH | 5 | 1±0.311 | p=0.2536 |
| | | | | NTG | 5 | 1.46±0.211 | |
| | | Tgfβ | Parametric Test (Unpaired T test) | VEH | 5 | 1±0.314 | \*p=0.031 |
| | | | | NTG | 5 | 5.78±1.803 | |

## Slide 7
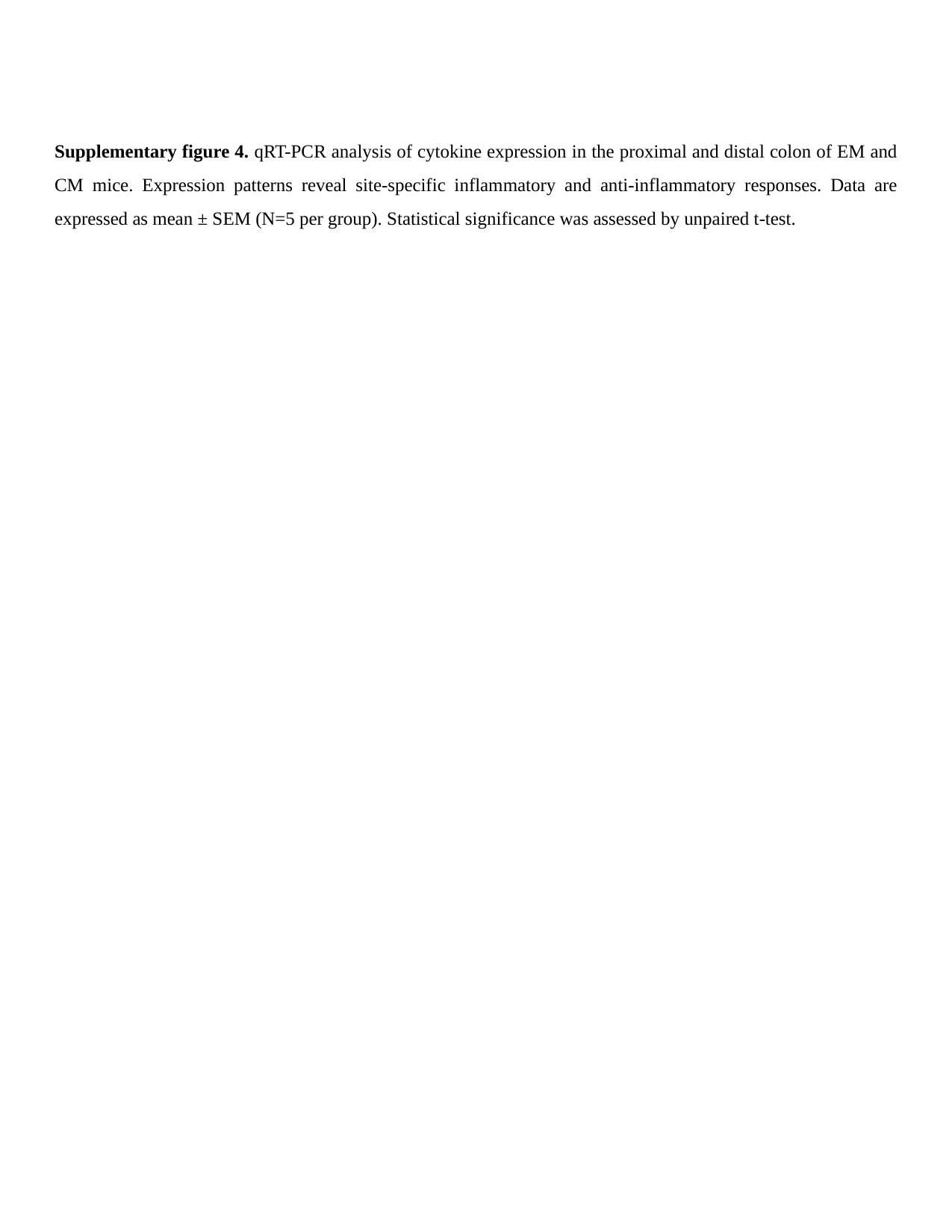

Supplementary figure 4. qRT-PCR analysis of cytokine expression in the proximal and distal colon of EM and CM mice. Expression patterns reveal site-specific inflammatory and anti-inflammatory responses. Data are expressed as mean ± SEM (N=5 per group). Statistical significance was assessed by unpaired t-test.

## Slide 8
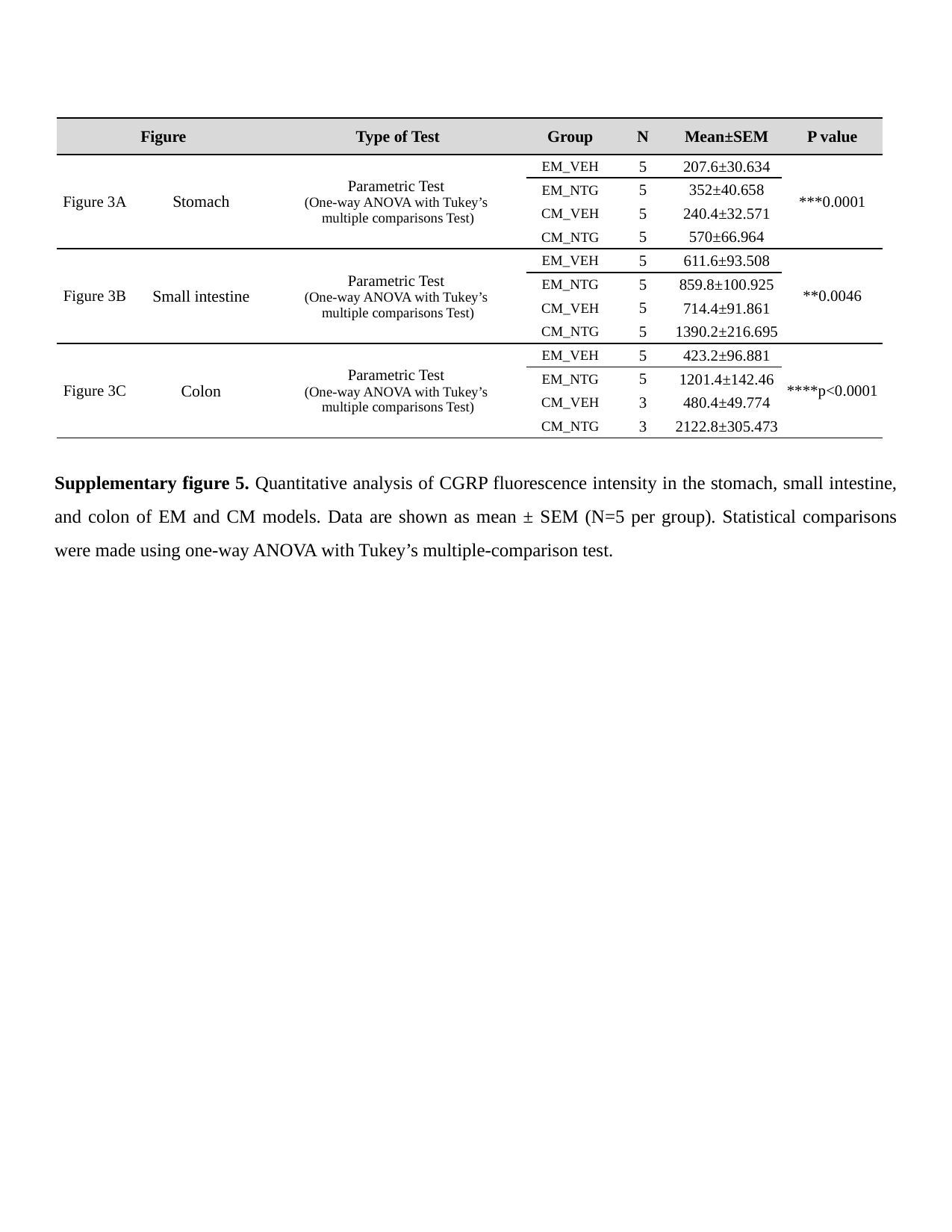

| Figure | | Type of Test | Group | N | Mean±SEM | P value |
| --- | --- | --- | --- | --- | --- | --- |
| Figure 3A | Stomach | Parametric Test (One-way ANOVA with Tukey’s multiple comparisons Test) | EM\_VEH | 5 | 207.6±30.634 | \*\*\*0.0001 |
| | | | EM\_NTG | 5 | 352±40.658 | |
| | | | CM\_VEH | 5 | 240.4±32.571 | |
| | | | CM\_NTG | 5 | 570±66.964 | |
| Figure 3B | Small intestine | Parametric Test (One-way ANOVA with Tukey’s multiple comparisons Test) | EM\_VEH | 5 | 611.6±93.508 | \*\*0.0046 |
| | | | EM\_NTG | 5 | 859.8±100.925 | |
| | | | CM\_VEH | 5 | 714.4±91.861 | |
| | | | CM\_NTG | 5 | 1390.2±216.695 | |
| Figure 3C | Colon | Parametric Test (One-way ANOVA with Tukey’s multiple comparisons Test) | EM\_VEH | 5 | 423.2±96.881 | \*\*\*\*p<0.0001 |
| | | | EM\_NTG | 5 | 1201.4±142.46 | |
| | | | CM\_VEH | 3 | 480.4±49.774 | |
| | | | CM\_NTG | 3 | 2122.8±305.473 | |
Supplementary figure 5. Quantitative analysis of CGRP fluorescence intensity in the stomach, small intestine, and colon of EM and CM models. Data are shown as mean ± SEM (N=5 per group). Statistical comparisons were made using one-way ANOVA with Tukey’s multiple-comparison test.

## Slide 9
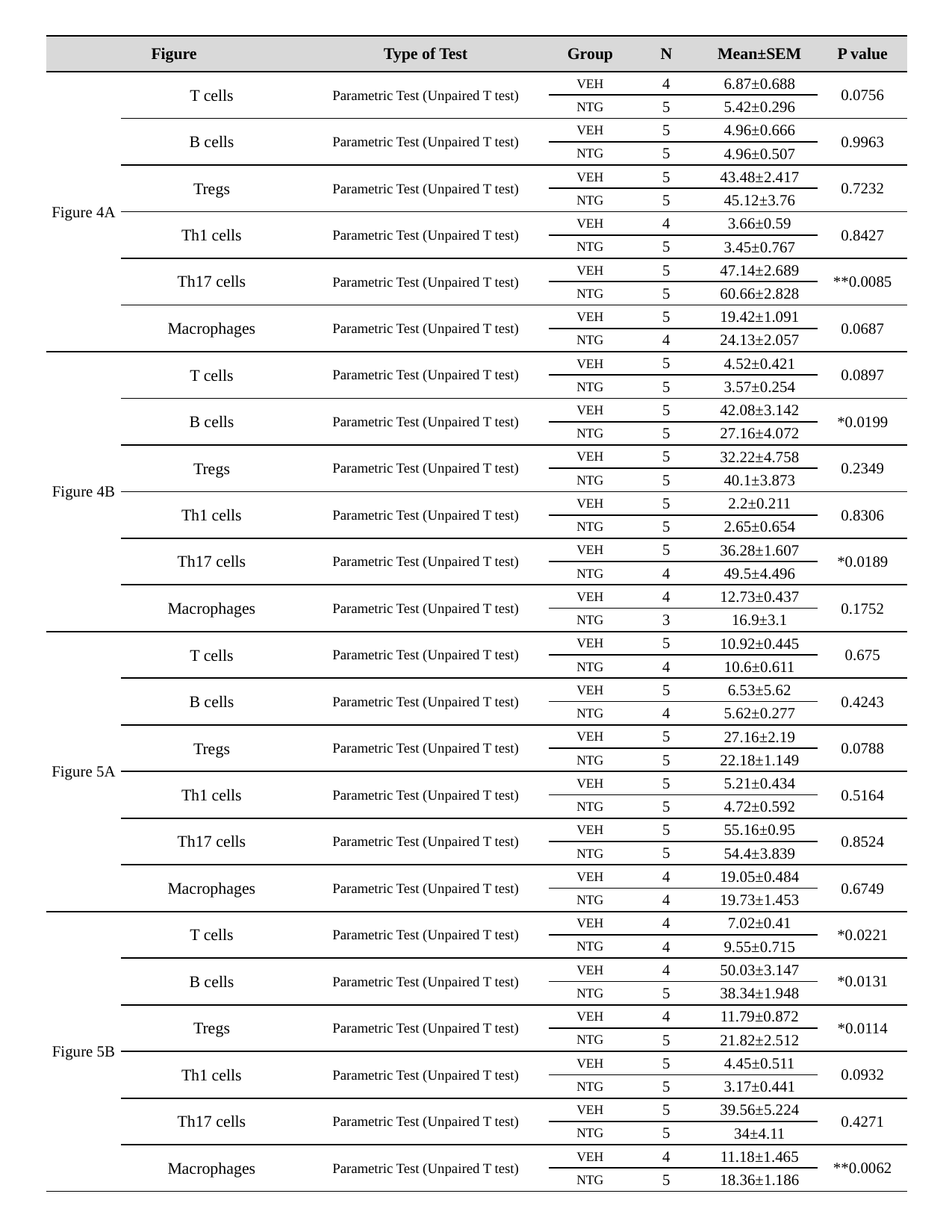

| Figure | | Type of Test | Group | N | Mean±SEM | P value |
| --- | --- | --- | --- | --- | --- | --- |
| Figure 4A | T cells | Parametric Test (Unpaired T test) | VEH | 4 | 6.87±0.688 | 0.0756 |
| | | | NTG | 5 | 5.42±0.296 | |
| | B cells | Parametric Test (Unpaired T test) | VEH | 5 | 4.96±0.666 | 0.9963 |
| | | | NTG | 5 | 4.96±0.507 | |
| | Tregs | Parametric Test (Unpaired T test) | VEH | 5 | 43.48±2.417 | 0.7232 |
| | | | NTG | 5 | 45.12±3.76 | |
| | Th1 cells | Parametric Test (Unpaired T test) | VEH | 4 | 3.66±0.59 | 0.8427 |
| | | | NTG | 5 | 3.45±0.767 | |
| | Th17 cells | Parametric Test (Unpaired T test) | VEH | 5 | 47.14±2.689 | \*\*0.0085 |
| | | | NTG | 5 | 60.66±2.828 | |
| | Macrophages | Parametric Test (Unpaired T test) | VEH | 5 | 19.42±1.091 | 0.0687 |
| | | | NTG | 4 | 24.13±2.057 | |
| Figure 4B | T cells | Parametric Test (Unpaired T test) | VEH | 5 | 4.52±0.421 | 0.0897 |
| | | | NTG | 5 | 3.57±0.254 | |
| | B cells | Parametric Test (Unpaired T test) | VEH | 5 | 42.08±3.142 | \*0.0199 |
| | | | NTG | 5 | 27.16±4.072 | |
| | Tregs | Parametric Test (Unpaired T test) | VEH | 5 | 32.22±4.758 | 0.2349 |
| | | | NTG | 5 | 40.1±3.873 | |
| | Th1 cells | Parametric Test (Unpaired T test) | VEH | 5 | 2.2±0.211 | 0.8306 |
| | | | NTG | 5 | 2.65±0.654 | |
| | Th17 cells | Parametric Test (Unpaired T test) | VEH | 5 | 36.28±1.607 | \*0.0189 |
| | | | NTG | 4 | 49.5±4.496 | |
| | Macrophages | Parametric Test (Unpaired T test) | VEH | 4 | 12.73±0.437 | 0.1752 |
| | | | NTG | 3 | 16.9±3.1 | |
| Figure 5A | T cells | Parametric Test (Unpaired T test) | VEH | 5 | 10.92±0.445 | 0.675 |
| | | | NTG | 4 | 10.6±0.611 | |
| | B cells | Parametric Test (Unpaired T test) | VEH | 5 | 6.53±5.62 | 0.4243 |
| | | | NTG | 4 | 5.62±0.277 | |
| | Tregs | Parametric Test (Unpaired T test) | VEH | 5 | 27.16±2.19 | 0.0788 |
| | | | NTG | 5 | 22.18±1.149 | |
| | Th1 cells | Parametric Test (Unpaired T test) | VEH | 5 | 5.21±0.434 | 0.5164 |
| | | | NTG | 5 | 4.72±0.592 | |
| | Th17 cells | Parametric Test (Unpaired T test) | VEH | 5 | 55.16±0.95 | 0.8524 |
| | | | NTG | 5 | 54.4±3.839 | |
| | Macrophages | Parametric Test (Unpaired T test) | VEH | 4 | 19.05±0.484 | 0.6749 |
| | | | NTG | 4 | 19.73±1.453 | |
| Figure 5B | T cells | Parametric Test (Unpaired T test) | VEH | 4 | 7.02±0.41 | \*0.0221 |
| | | | NTG | 4 | 9.55±0.715 | |
| | B cells | Parametric Test (Unpaired T test) | VEH | 4 | 50.03±3.147 | \*0.0131 |
| | | | NTG | 5 | 38.34±1.948 | |
| | Tregs | Parametric Test (Unpaired T test) | VEH | 4 | 11.79±0.872 | \*0.0114 |
| | | | NTG | 5 | 21.82±2.512 | |
| | Th1 cells | Parametric Test (Unpaired T test) | VEH | 5 | 4.45±0.511 | 0.0932 |
| | | | NTG | 5 | 3.17±0.441 | |
| | Th17 cells | Parametric Test (Unpaired T test) | VEH | 5 | 39.56±5.224 | 0.4271 |
| | | | NTG | 5 | 34±4.11 | |
| | Macrophages | Parametric Test (Unpaired T test) | VEH | 4 | 11.18±1.465 | \*\*0.0062 |
| | | | NTG | 5 | 18.36±1.186 | |

## Slide 10
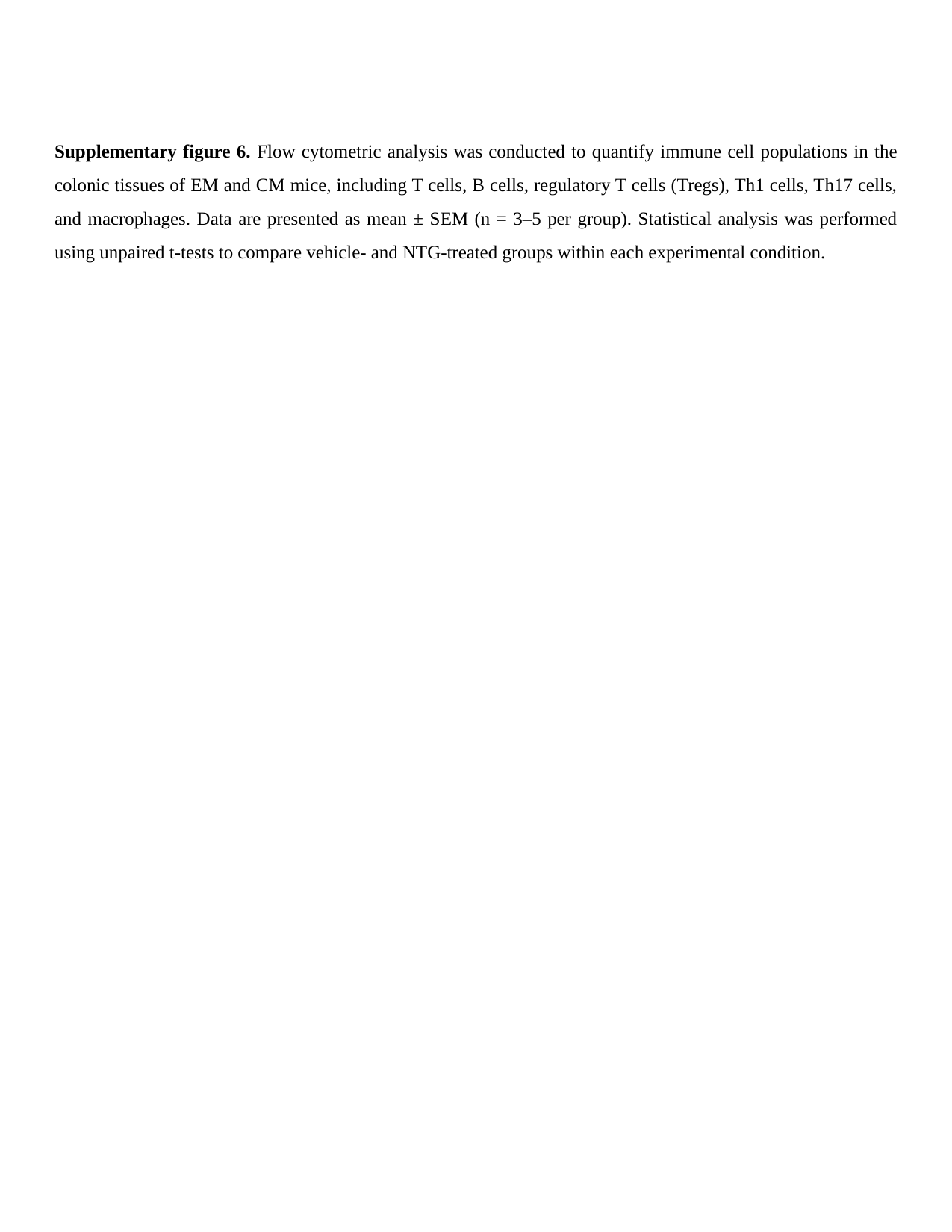

Supplementary figure 6. Flow cytometric analysis was conducted to quantify immune cell populations in the colonic tissues of EM and CM mice, including T cells, B cells, regulatory T cells (Tregs), Th1 cells, Th17 cells, and macrophages. Data are presented as mean ± SEM (n = 3–5 per group). Statistical analysis was performed using unpaired t-tests to compare vehicle- and NTG-treated groups within each experimental condition.
