# Supplementary material for: Distinct Gut–Brain Axis Dysregulation in Episodic Versus Chronic Migraine: Insights from NTG-Induced Mouse Models
Source: Int J Mol Sci. 2025 Oct 29;26(21):10493. doi: 10.3390/ijms262110493 (PMC12607510; doi:10.3390/ijms262110493)
Supplement: Supplementary file 1 [file ijms-26-10493-s001.zip › Supplementary Materials.pdf]

Supplementary figure 1. Statistical analysis of gastrointestinal length measurements (stomach, small intestine, and colon) and changes in food intake and body weight in EM and CM models. Data are expressed as mean ± SEM (N=5 per group). Statistical analysis was performed using one-way or two-way ANOVA with Tukey's or Sidak's post-hoc tests, respectively.

Supplementary figure 2. qRT-PCR analysis of cytokine expression (IL-1β, IL-6, IL-8, TNF-α, IL-4, IL-10, and TGF-β) in the stomach of EM and CM mice following NTG administration. Data represent mean ± SEM (N=5 per group). Statistical significance was determined using unpaired t-tests.

Supplementary figure 3. qRT-PCR analysis of cytokine expression in the duodenum and ileum of EM and CM mice. Cytokines analyzed include IL-1β, IL-6, IL-8, TNF-α, IL-4, IL-10, and TGF-β. Data are presented as mean ± SEM (N=5 per group). Parametric tests (unpaired t-tests) were used for comparisons.

Supplementary figure 4. qRT-PCR analysis of cytokine expression in the proximal and distal colon of EM and CM mice. Expression patterns reveal site-specific inflammatory and anti-inflammatory responses. Data are expressed as mean ± SEM (N=5 per group). Statistical significance was assessed by unpaired t-test.

Supplementary figure 5. Quantitative analysis of CGRP fluorescence intensity in the stomach, small intestine, and colon of EM and CM models. Data are shown as mean ± SEM (N=5 per group). Statistical comparisons were made using one-way ANOVA with Tukey's multiple-comparison test.

Supplementary figure 6. Flow cytometric analysis was conducted to quantify immune cell populations in the colonic tissues of EM and CM mice, including T cells, B cells, regulatory T cells (Tregs), Th1 cells, Th17 cells, and macrophages. Data are presented as mean ± SEM (n = 3–5 per group). Statistical analysis was performed using unpaired t-tests to compare vehicle- and NTG-treated groups within each experimental condition.
